# HLA class I loss in metachronous metastases prevents continuous T cell recognition of mutated neoantigens in a human melanoma model

**DOI:** 10.18632/oncotarget.16048

**Published:** 2017-03-09

**Authors:** Barbara Schrörs, Silke Lübcke, Volker Lennerz, Martina Fatho, Anne Bicker, Catherine Wölfel, Patrick Derigs, Thomas Hankeln, Dirk Schadendorf, Annette Paschen, Thomas Wölfel

**Affiliations:** ^1^ Internal Medicine III, University Cancer Center (UCT) and Research Center for Immunotherapy (FZI), University Medical Center (UMC) of the Johannes Gutenberg University and German Cancer Consortium (DKTK), Partner Site Frankfurt/Mainz, Mainz, Germany; ^2^ Institute for Molecular Genetics, Johannes Gutenberg University, Mainz, Germany; ^3^ Department of Dermatology, University Hospital, University Duisburg-Essen and German Cancer Consortium (DKTK), Partner Site Essen/Düsseldorf, Essen, Germany

**Keywords:** melanoma, mutated neoantigens, high-throughput sequencing, immune escape, HLA class I loss

## Abstract

T lymphocytes against tumor-specific mutated neoantigens can induce tumor regression. Also, the size of the immunogenic cancer mutanome is supposed to correlate with the clinical efficacy of checkpoint inhibition. Herein, we studied the susceptibility of tumor cell lines from lymph node metastases occurring in a melanoma patient over several years towards blood-derived, neoantigen-specific CD8^+^ T cells. In contrast to a cell line established during early stage III disease, all cell lines generated at later time points from stage IV metastases exhibited partial or complete loss of HLA class I expression. Whole exome and transcriptome sequencing of the four tumor lines and a germline control were applied to identify expressed somatic single nucleotide substitutions (SNS), insertions and deletions (indels). Candidate peptides encoded by these variants and predicted to bind to the patient's HLA class I alleles were synthesized and tested for recognition by autologous mixed lymphocyte-tumor cell cultures (MLTCs). Peptides from four mutated proteins, HERPUD1^G161S^, INSIG1^S238F^, MMS22L^S437F^ and PRDM10^S1050F^, were recognized by MLTC responders and MLTC-derived T cell clones restricted by HLA-A*24:02 or HLA-B*15:01. Intracellular peptide processing was verified with transfectants. All four neoantigens could only be targeted on the cell line generated during early stage III disease. HLA loss variants of any kind were uniformly resistant. These findings corroborate that, although neoantigens represent attractive therapeutic targets, they also contribute to the process of cancer immunoediting as a serious limitation to specific T cell immunotherapy.

## INTRODUCTION

Immune checkpoint inhibitors elicit impressive responses in a series of tumor entities [1]. There is growing evidence that tumors with a high mutation load are particularly responsive to checkpoint blockade [2–5]. Indeed, checkpoint inhibitors can boost T cell reactivity against neoantigens generated by somatic mutations [6]. However, a preexisting anti-tumoral T cell immunity is required for these immunomodulatory antibodies to show an effect [7]. Neoantigens represent optimal targets as they are truly tumor-specific, which prevents autoimmune responses, and they are lacking immunological tolerance [8]. The therapeutic potential of targeting mutated antigens has been shown before in mice [9, 10] and men [11, 12], and it has been reported that responses against neoantigens can occur at high frequencies among circulating T cells or even dominate the anti-tumoral T cell repertoire [13–15]. CD8^+^ T cells appear to play a key role. They can directly recognize and lyse tumor cells and tumor infiltration with CD8^+^ T cells was shown to be associated with better prognosis [16]. Using exome sequencing, several groups succeeded in identifying mutated tumor antigens in individual patients during the recent years [6, 8, 17–19].

Herein, we searched for potentially immunogenic single nucleotide substitutions (SNS) and insertions and deletions (indels) using exome and transcriptome sequencing data generated from four melanoma cell lines derived from distinct lymph node (LN) metastases of patient Ma-Mel-86 [20] and from an autologous lymphoblastoid cell line as germline control. By this means, 181 expressed non-synonymous somatic SNS and ten expressed non-synonymous indels were identified in the four melanoma cell lines. Using autologous mixed lymphocyte-tumor cell cultures (MLTCs) and T cell clones derived thereof, four mutations were found to encode patient-specific HLA class I-restricted neoantigens. Notably, tumor cell lines derived from late occurring metastases did not present the mutated antigens.

## RESULTS

### Melanoma model Ma-Mel-86

The human melanoma model was derived from patient Ma-Mel-86. Her clinical course is summarized in Supplementary Figure 1 and detailed in [20]. Permanent melanoma cell lines Ma-Mel-86a, −86b, −86c and −86f were established from four distinct LN metastases occurring during stage III disease in 01/2002 and during stage IV disease in 02/2004, 05/2005 and 12/2008, respectively. Ma-Mel-86b and −86f represented HLA class I loss variants due to a biallelic beta-2-microglobulin gene (*B2M*) inactivation. Ma-Mel-86c was lacking one complete HLA class I haplotype [20]. Autologous peripheral blood mononuclear cells (PBMCs) were isolated in 05/2002, 04/2004 and 08/2004. The model system provided a rare opportunity to analyze T cell reactivity against somatic mutations throughout the course of disease.

### Sequencing results

For a systematic identification of immunogenic SNS and indels, we performed whole exome and transcriptome sequencing on the four melanoma cell lines and on autologous EBV-B cells as germline control. Considering biological variance, we sequenced DNA duplicates and RNA triplicates isolated from separate cultures and two different time points each (Supplementary Figure 2A). Replicates were processed in parallel (Supplementary Figure 2B). In the exome data, a mean coverage of 49.3-fold was achieved with on average 82.7% of the targeted bases being covered by at least 15 reads (Table 1). The alignment of the transcriptome reads resulted in an average of 91.2% uniquely mapped reads.

**Table 1:** Metrics of the exome and transcriptome sequencing

Cell line	Replicate	Type	Quality filter passing reads	Mean exome coverage[−fold]	Number of putative somatic mutations
Ma-Mel-86a	1	Exome	51,085,483	39.9	2,099
	2		51,368,377	39.9	
	1	Transcriptome	77,792,234	−	5,992
	2		68,766,926	−	
	3		84,137,830	−	
Ma-Mel-86b	1	Exome	51,665,217	39.8	2,504
	2		65,238,045	51.1	
	1	Transcriptome	80,666,672	−	8,974
	2		105,562,290	−	
	3		104,192,306	−	
Ma-Mel-86c	1	Exome	63,704,508	48.7	2,236
	2		79,512,089	61.8	
	1	Transcriptome	59,468,122	−	2,914
	2		42,750,918	−	
	3		33,877,483	−	
Ma-Mel-86f	1	Exome	67,931,163	48.7	3,108
	2		72,904,674	52.5	
	1	Transcriptome	55,071,615	−	4,074
	2		36,056,083	−	
	3		59,223,897	−	
Ma-Mel-86-EBV-B	1	Exome	59,068,661	45.8	−
	2		83,437,818	64.9	
	1	Transcriptome	80,424,119	−	−
	2		26,741,378	−	
	3		75,696,328	−	

Alterations compared to the reference genome were detected and mutations with an error probability of more than 5% (i.e. Phred score < 13) and a coverage of less than five transcriptome or ten exome reads were rejected. Applying these criteria, 72–74% of the exomic variants were detected in both replicates and 56–64% of the variants found in the transcriptomes were confirmed by at least a second transcriptomic replicate. The variants detected in both exomes and at least two transcriptomes of a tumor cell line accounted on average for about 10% of all variants detected in a tumor cell line.

Somatic mutations were located by subtraction of the variants found in the autologous EBV-B cells. Per melanoma cell line, 2,099–3,108 somatic mutations consisting of SNS and indels were detected in both exome replicates (Table 1) and about 85% of these somatic variants were SNS. 46–56% of the somatic SNS were cytosine-to-thymine transitions typically indicating UV-induced DNA damage [21]. In the transcriptomes, a higher total number of putative somatic mutations (2,914–8,974) was determined. For the final selection of potentially immunogenic SNS in the transcriptomes only those were considered as somatic mutations for which only wild-type alleles were detected in the germline control. However, in some cases, the mutated site was not covered in the EBV-B data. Among these variants only those were included in further analysis for which at least one of the melanoma cell lines carried only wild-type alleles.

**Figure 1:**
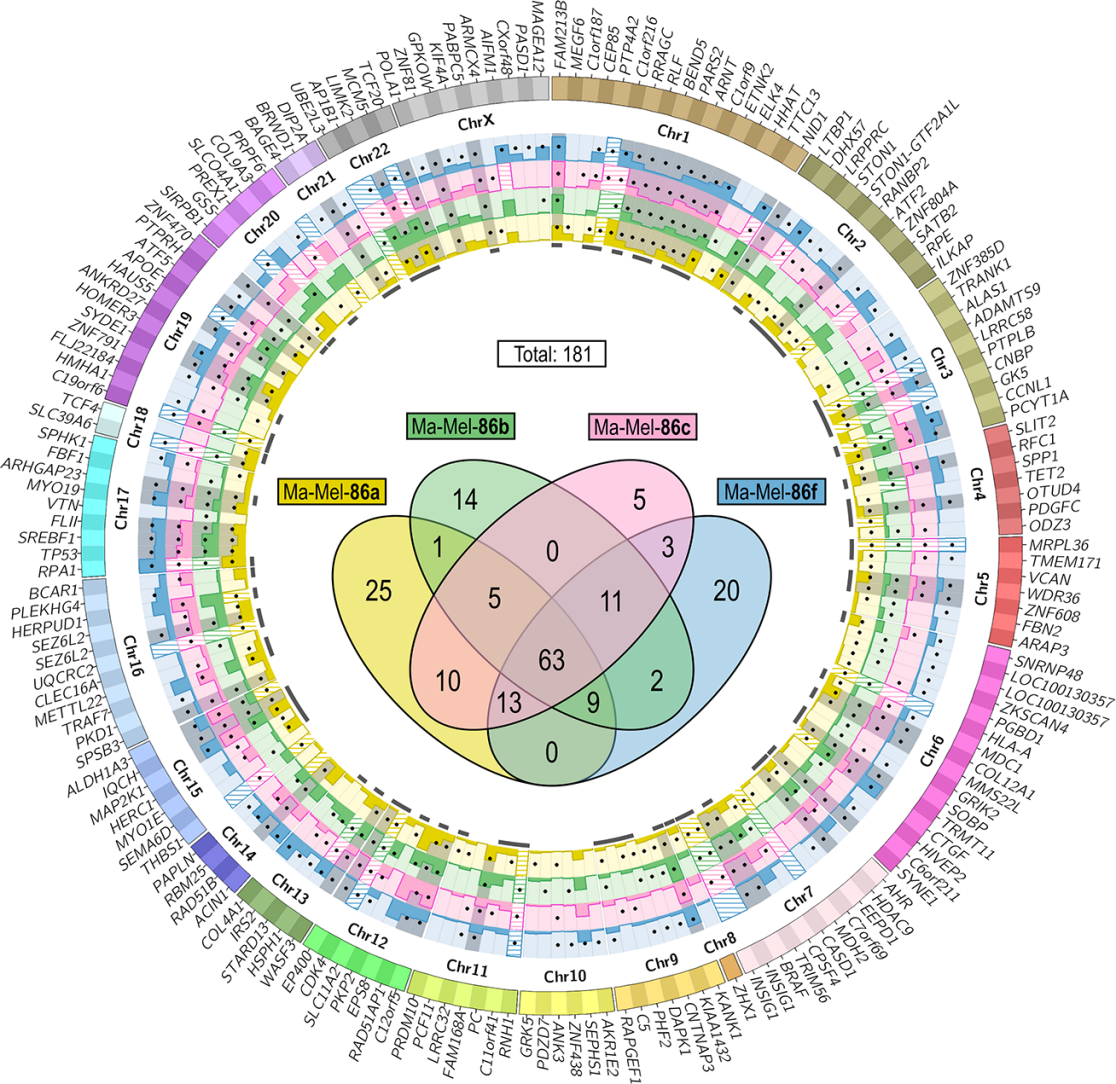
Localization of potentially immunogenic mutations in Ma-Mel-86 Filtering according to the scheme in Supplementary Figure 2B resulted in a list of 181 somatic expressed non-synonymous SNS. Their distribution to the four melanoma cell lines is depicted in the Venn diagram (center). The 178 affected genes are listed on the outer ring of the Circos plot sorted by their genomic coordinates. Genes listed twice carried two different mutations (*LOC100130357* und *SEZ6L2*) or two isoform-specific amino acid exchanges caused by the same mutation (*INSIG1*). Gene expression levels are given as normalized read counts (means of three replicates) in the bar graphs determined from the RNA-seq data of Ma-Mel-86a (yellow), −86b (green), −86c (magenta) and −86f (blue). Expression values above 2,000 are indicated with shading. Black dots on the bar graphs mark which cell line carried the mutations and mutations present in all four tumor cell lines are gray-shaded. Marks below the bar graphs indicate which SNS led to at least one peptide candidate with a predicted HLA class I binding affinity of ≤ 500 nM (NetMHC) and a percentile rank of ≤ 10 (IEDB).

The identified putative somatic mutations confirmed by at least a second replicate of the same sequencing procedure were filtered for non-synonymous SNS in protein-coding regions. Comparison of the SNS lists from the exome and transcriptome of each melanoma cell line and manual curation with the Integrative Genomics Viewer (IGV) [22] validated 181 of 244 non-synonymous SNS detected in the transcriptomes as somatic as described above. Remarkably, one third (61) of these SNS had been rejected from the exome data due to a too low coverage. As depicted in the Venn diagram in Figure 1, 63 of the SNS were common to all four tumor cell lines, while the remaining were heterogeneously distributed. Thirty-six SNS were only expressed by the HLA class I loss variants Ma-Mel-86b or −86f.

Only 2.5–3.8% of the somatic indels observed in the melanoma cell lines in both exome replicates induced changes in the amino acid sequence. This corresponded to 17 different non-synonymous indels in total. For ten of them, at least 20 supporting reads could be detected in the transcriptome data of at least one melanoma cell line. These indels were assumed to be expressed on mRNA level. Only one insertion was specific for an HLA class I-negative melanoma cell variant (Ma-Mel-86b).

### Prediction of HLA-binding peptides from selected non-synonymous somatic SNS and indels

For each of the 145 SNS and nine indels detectable in at least one of the HLA class I-expressing cell lines Ma-Mel-86a and −86c, the respective proteins were screened for mutated peptides that could be presented by the patient's HLA class I alleles. In total, 207 SNS-derived and 30 indel-derived octa-, nona- and decamers were predicted to bind to at least one of the six HLA class I alleles with a binding affinity of ≤500 nM according to NetMHC 3.4 [23] and a percentile rank of ≤10 as calculated with the IEDB MHC class I-binding prediction tool [24]. Some of these peptides originated from splice variants that were not expressed as at least one of the peptide-encoding exons lacked coverage in the patient's RNA-seq data and were excluded. Furthermore, we decided to test only indel-derived nona- and decamers. Thus, 174 SNS-derived and 22 indel-derived peptides (Supplementary Table 1) were finally selected. They were encoded by 80 different somatic non-synonymous SNS (marks below bar graphs in Figure 1) in 79 distinct genes and six different somatic non-synonymous indels in six distinct genes. Eleven of the peptides were encoded by seven SNS that were present in Ma-Mel-86c, but not in −86a, and that were predicted to bind to the HLA class I alleles not expressed by Ma-Mel-86c cells due to the haplotype loss. In the following, these peptides are referred to as 86c^NP^ peptides (NP: not presented). In order to stimulate T cells against 86c^NP^ peptides, we generated three stable Ma-Mel-86c transfectants that were reconstituted with the HLA class I alleles lost in Ma-Mel-86c.

### Immunogenicity of the selected synthetic octa-, nona- and decamer peptides

**Figure 2:**
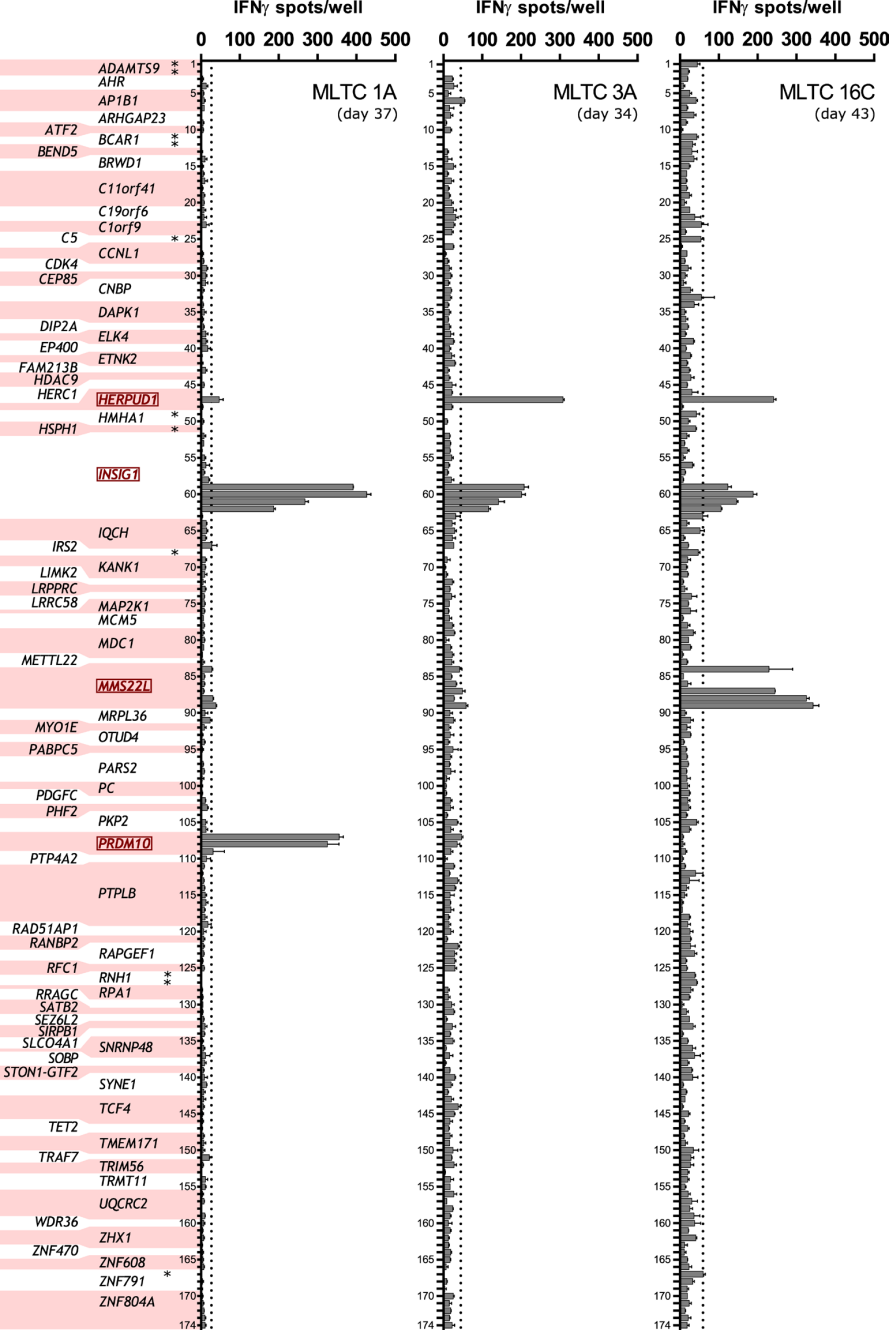
Peptide recognition by autologous MLTC responder populations One hundred seventy-four selected SNS-encoded synthetic peptides (numbered and allocated to the respective coding mutated gene, peptide sequences are listed in Supplementary Table 1) were tested for their recognition by CD8^+^ responder lymphocytes of eight independent MLTCs (see Table 2) in IFNγ ELISpot assays. Peptide reactivity was observed with three MLTCs. These were MLTC 1A (day 37, 30,000 cells/well), 3A (day 34, 23,000 cells/well) and 16C (day 43, 30,000 cells/well). Shown are mean spot counts of duplicates (error bars indicate standard errors of the mean, SEM). Peptides were added at a concentration of 10 μg/ml in the presence (MLTC 3A) or absence (all other MLTCs) of 6×10^4^ 293T cells that had been electroporated with the patient's HLA class I alleles one day before. 86C^NP^ peptides are marked with asterisks in the axis label of the left panel and were only tested with MLTC 16C. Background reactivities were determined in the absence of peptides (not shown) and peptides inducing at least mean background counts plus three SD (dotted lines) were defined as positive. Mutated genes encoding recognized peptides are marked in red.

Eight independent MLTCs were generated from PBMCs or CD8^+^ T cells isolated from blood donated by the patient (Table 2). The lymphocytes were stimulated with the melanoma cell lines Ma-Mel-86a (MLTCs 1A, 3A and 4A), −86c (MLTCs 1C, 2C, 7C and 15C) or with HLA transfectants of Ma-Mel-86c (MLTC 16C). After three to eight weekly stimulations (Table 2), CD8^+^ MLTC responders were tested with IFNγ ELISpot assays for recognition of the 196 mutated candidate peptides (Supplementary Table 1). 86c^NP^ peptides were analyzed with MLTC 16C responders only and indel-derived peptides could not be tested with MLTC 15C and MLTC 1A due to lack of material. In total, we found eleven SNS-derived peptides to be reproducibly recognized by up to three of the MLTCs with average spot counts above the threshold (mean background count plus three SD, Figure 2). The responsive MLTCs 1A, 3A and 16C had been stimulated with either Ma-Mel-86a or HLA-reconstituted Ma-Mel-86c. None of the 86c^NP^ peptides were recognized (Figure 2). None of the MLTCs stimulated with unmodified Ma-Mel-86c cells showed peptide reactivity (not shown). And no reactivity was observed against any of the indel-derived peptides (not shown).

**Table 2:** MLTC responder populations used for the analysis of the immunogenicity of the selected peptide candidates

MLTC	generated from	Blood donation	Stimulator cells	Culture time at peptide-reactivity test(days)
**1A**	PBMCs	08/2004	Ma-Mel-86a	37
**3A**	CD8*^+^*	04/2004	Ma-Mel-86a	34
**4A**	PBMCs	05/2002	Ma-Mel-86a	62
**1C**	PBMCs	08/2004	Ma-Mel-86c	28
**2C**	CD8*^+^*	08/2004	Ma-Mel-86c	29
**7C**	CD8*^+^*	08/2004	Ma-Mel-86c	40
**15C**	CD8*^+^*	08/2004	Ma-Mel-86c	28
**16C**	CD8*^+^*	04/2004	Ma-Mel-86c + HLA-A*24:02Ma-Mel-86c + HLA-B*15:01Ma-Mel-86c + HLA-C*03:03	43

The immunogenic peptides were encoded by the four mutated genes *HERPUD1*^G161S^ (homocysteine-inducible, endoplasmic reticulum stress-inducible, ubiquitin-like domain member 1), *INSIG1*^S238F^ (insulin-induced gene 1), *MMS22L*^S437F^ (MMS22-like, DNA repair protein) and *PRDM10*^S1050F^ (PR domain containing 10) as listed in Table 3. In case of INSIG1^mut^, for which peptide candidates encoded by two distinct isoforms were tested, only peptides derived from isoform 1 were recognized. For MMS22L^mut^, INSIG1^mut^ and PRDM10^mut^, we observed reactivity against two to four peptides that varied in length only. Among the four antigens, only HERPUD1^mut^ had been identified independently via cDNA library expression screening with MLTC 1A responders (S. Lübcke, unpublished material).

**Table 3:** Characteristics of mutated Ma−Mel−86 neoantigens recognized by autologous CD8^+^ T cells

Mutated neoantigen (amino acid exchange)	Codon change (wt/mut)	HLA restriction	Peptide sequence^a^	Mutations present and expressed in Ma−Mel−86 cell lines^b^				Recognized by MLTC			Reactivity against Ma−Mel−86 cell lines^c^			
				86a	86b	86c	86f	1A	3A	16C	86a	86b	86c	86f
*PRDM10*^mut^_1042-1050_ (S1050F)	t**C**c/t**T**c	A*24:02	TYLPSAWN**F**	+	−	−	−	×	×		+	−	−	−
*INSIG1*^mut^_233-241_ (S238F)	t**C**c/t**T**c	A*24:02	VYQYT**F**PDF	+	+	+	−	×	×	×	+	−	−	−
*MMS22L* ^mut^_429-438_ (S437F)	t**C**c/t**T**c	A*24:02	YYSKNLNS**F**F	+	+	+	+		×	×	+	−	−	−
*HERPUD1*^mut^_154-162_ (G161S)	**G**gt/**A**gt	B*15:01	GLGPGFS**S**Y	+	−	+	−	×	×	×	+	−	−	−

a: peptide sequences were deduced from truncation experiments, testing of synthetic peptides and HLA class I binding predictions (mutated residue underlined and bold), b: as detected in at least two of three RNA−seq replicates and at least one of two exome replicates, c: as determined with clonal T cells against the mutated neoantigens.

### Generation of peptide-specific CD8^+^ T cell clones

MLTCs 1A (day 35), 1A (day 45), 3A (day 32) and 16C (day 41), all consisting of at least 90% CD8^+^ T cells, were cloned by limiting dilution. After three to four weeks, clones were tested with IFNγ ELISpot assays for peptide recognition. Four of 123 T cell clones derived from MLTC 1A (day 45) recognized HERPUD1^mut^. INSIG1^mut^-directed T cell clones could be expanded from all three MLTCs [1A (day 35): 32 of 67 T cell clones, 3A: 6 of 276 T cell clones, 16C: 4 of 124 T cell clones]. Clonal T cells targeting PRDM10^mut^ were derived only from MLTC 1A (day 35; 14 of 67) and MMS22L^mut^-reactive T cell clones were isolated from MLTC 16C (17 of 124). T cell clones 1A/39 (anti-HERPUD1^mut^), 3A/115 (anti-INSIG1^mut^), 1A/1003 (anti-PRDM10^mut^) and 16C/114 (anti-MMS22L^mut^) were selected for further functional analyses.

The TCR Vß cDNA sequences of two T cell clones were determined for each neoantigen specificity. While T cell clones against HERPUD1^mut^, PRDM10^mut^ and MMS22L^mut^ carried identical TCRß clonotypes, two distinct CDR3ß regions were identified for T cell clones against INSIG1^mut^ (Supplementary Table 3).

### Confirmation of intracellular peptide processing and presentation

**Figure 3:**
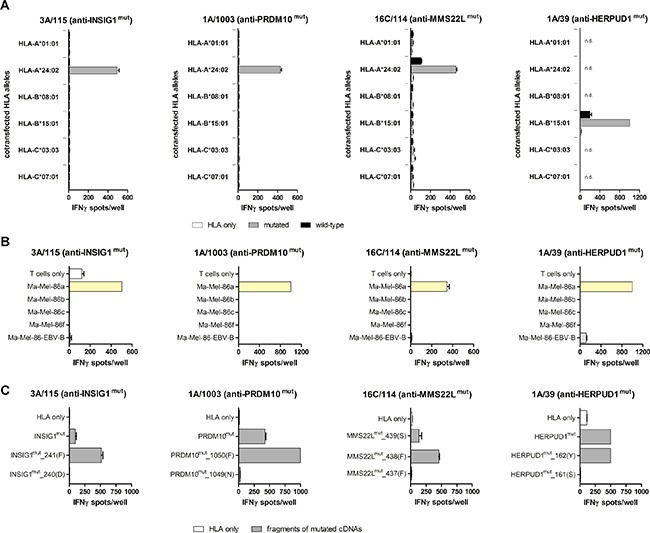
Characterization of neoantigen recognition (**A**) Mutated or wild-type antigen-coding cDNAs (300 ng/well) and cDNAs encoding the patient's HLA class I alleles (100 ng/well) were transfected into COS-7 cells (*INSIG1*, *PRDM10* and *MMS22L*) or 293T cells (*HERPUD1*) (each 20,000 cells/well), and transfectants were tested in IFNγ ELISpot assays for recognition by MLTC-derived, neoantigen-specific T cell clones (3A/115 anti-INSIG1^mut^ and 1A/1003 anti-PRDM10^mut^: 20,000 effector cells/well, 16C/114 anti-MMS22L^mut^: 44,000 effector cells/well, 1A/39 anti-HERPUD1^mut^: 10,000 effector cells/well). Data represent mean spot counts of duplicates (error bars: SEM). They are representative of at least two independent experiments. Abbreviation: n.d., not done. (**B**) Recognition of autologous tumor cell lines and Ma-Mel-EBV-B cells (each 50.000 cells/well) by neoantigen-specific T cell clones was determined under assay conditions described in (A). (**C**) Full-length antigen-encoding cDNAs and 3′-fragments of the cDNAs were transfected into COS-7 or 293T cells together with the restricting HLA alleles HLA-A*24:02 (for INSIG1, PRDM10 and MMS22L) or HLA-B*15:01 (for HERPUD1) and the recognition of the transfectants was tested under the conditions described in (A), but with 30,000 effector cells/well in case of T cell clone 16C/114 (anti-MMS22L^mut^). cDNA fragment names indicate the number of the C-terminal codon and amino acid in parentheses.

Peptide-reactive T cell clones were tested for their ability to recognize COS-7 or 293T cells transfected with mutated or wild-type cDNAs together with cDNAs encoding the patient's HLA class I alleles (Figure 3A). The recognition of HERPUD1^mut^ was restricted by HLA-B*15:01 as already known from cDNA library expression screening (S. Lübcke, unpublished). cDNAs encoding INSIG1^mut^, MMS22L^mut^ and PRDM10^mut^ were recognized only upon cotransfection of HLA-A*24:02. A weak reactivity was observed against targets cotransfected with *MMS22L*^wt^ and HLA-A*24:02-cDNA as well as *HERPUD1*^wt^ and HLA-B*15:01-cDNA. The detected spot counts, however, were about 5-fold lower than those achieved with the respective mutated cDNAs under the same expression conditions. There was no or only weak and irreproducible recognition of autologous EBV-B cells naturally expressing wild-type *MMS22L* and wild-type *HERPUD1* (Figure 3B), which indicated that overexpression of the wild-type cDNAs was required to induce T cell reactivity.

Table 3 summarizes the distribution and recognition patterns of the mutated antigens among the patient's melanoma cell lines. Although *HERPUD1*, *INSIG1* and *MMS22L* were mutated in more than one of the melanoma cell lines, only Ma-Mel-86a cells were recognized by the respective T cell clones (Figure 3B). The determined HLA restrictions confirmed the predictions of the algorithms and explained the exclusive recognition of Ma-Mel-86a as HLA-A*24:02 and HLA-B*15:01 were not expressed by the other melanoma cell lines.

### Determination of the C-terminal peptide ends

Due to their low purity, testing of the synthetic peptides could not provide reliable information about the minimal length of the immunogenic peptides. To identify the C-termini of the mutated peptides, 3‘-deletion fragments were generated from cDNAs encoding HERPUD1^mut^, INSIG1^mut^, PRDM10^mut^ and MMS22L^mut^. The cDNA fragments were co-transfected with the restricting HLA allele into COS-7 or 293T cells and transfectants were tested for recognition by the respective neoantigen-specific T cell clones (Figure 3C). C-terminal residues critical for recognition were phenylalanine (F) on position 241 for INSIG1^mut^, the mutated residue phenylalanine (F) on position 1050 for PRDM10^mut^, phenylalanine (F) on position 438 for MMS22L^mut^, and tyrosine (Y) on position 162 for HERPUD1^mut^. These data proved that the mutated amino acid residues were integral components of the respective immunogenic peptides for all four neoantigens. Hence, based on the recognized peptides, the cDNA fragmentation experiments and the predicted HLA class I binding affinity, we postulated the peptide-coding regions and peptides listed in Table 3.

### Low frequency of neoantigen-specific T cells in the patient's peripheral blood

We performed a standardized *ex vivo* IFNγ ELISpot assay [25] with PBMCs from 08/2004. The detection limit was assumed to be 5 in 10^5^ PBMCs with an at least twofold increase of spot counts over background [26]. However, there was no reactivity detectable against any of the four neoantigens (data not shown). Therefore, deep sequencing of TCRβ CDR3 regions was performed on PBMCs collected in 05/2002 and in 08/2004 (Supplementary Table 3). The clonotypes of T cells against all four mutated neoantigens were detectable in the 08/2004 sample. The frequencies of the neoantigen-specific T cells were consistently below 4 clonotypic reads per 10^5^ productive rearrangements (HERPUD1^mut^: 1.6, MMS22L^mut^: 1.3, PRDM10^mut^: 0.7, INSIG1^mut^: 0.2 and 3.8). This explained the failure to detect the T cells in the *ex vivo* ELISpot assay. In the 05/2002 blood sample, only the clonotype against MMS22L^mut^ was detected at a frequency of 6.1 clonotypic reads per 10^5^ productive rearrangements.

### Expression levels of mRNAs encoding mutated neoantigens

**Figure 4:**
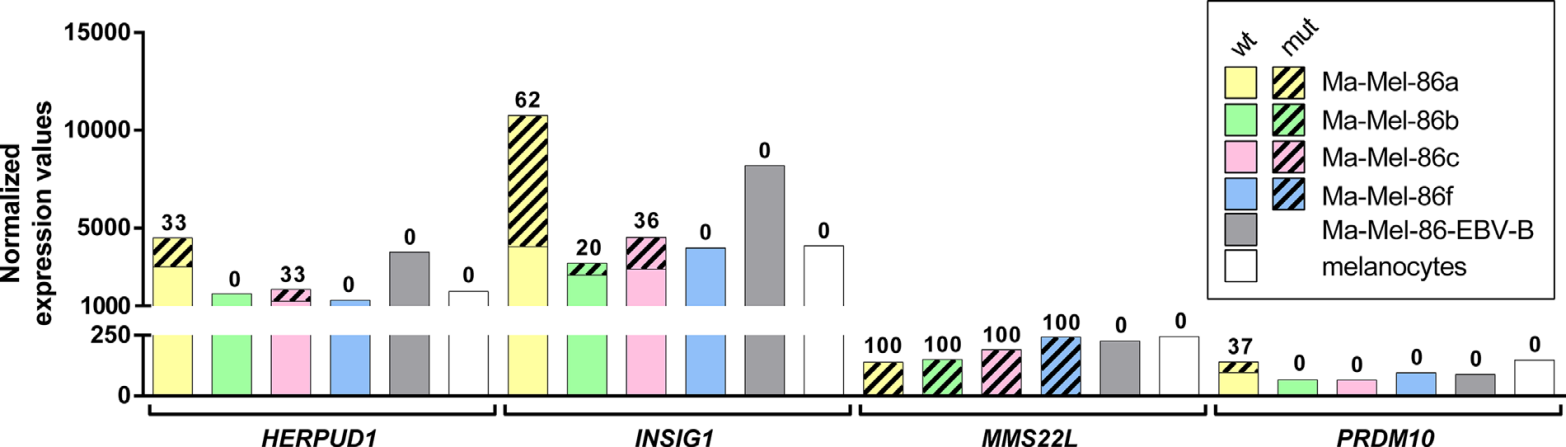
mRNA expression levels of the genes harboring immunogenic mutations Mean normalized expression values of three RNA-seq replicates per melanoma cell line and germline control Ma-Mel-86-EBV-B are depicted in comparison to a publicly available melanocyte data set (no replicates). The mean proportion of the mutated allele is visualized by shading and the corresponding percentages are given on top of the bars.

We determined the expression levels of the mutated mRNAs in the generated RNA-seq data in comparison to publicly available data of melanocytes [27]. In Ma-Mel-86a, *INSIG1* was found to be higher expressed than *HERPUD1* (Figure 4). 62% of the reads aligning to the mutated genomic coordinate in *INSIG1* were derived from the mutated allele. In case of *HERPUD1*, the wild-type allele was dominating and only 33% of the aligned reads contained the mutation. However, *MMS22L* and *PRDM10* were expressed at much lower levels. The wild-type allele of *MMS22L* was detected in none of the four melanoma cell lines. The expression level of *PRDM10* in Ma-Mel-86a and −86f was comparable to the reference melanocytes, whereas it was lower in Ma-Mel-86b and −86c. This gene was only mutated in Ma-Mel-86a and 37% of the reads aligning to the mutated position carried the SNS.

## DISCUSSION

Whole exome and transcriptome sequencing was applied to identify neoantigens encoded by somatic non-synonymous SNS and indels in the human melanoma model Ma-Mel-86. While exome sequencing has been used by others for the same purpose [6, 8, 17–19], transcriptome sequencing has so far mainly served to validate the expression of the mutations detected by exome sequencing. Remarkably, we initially detected 61 of 181 somatic non-synonymous SNS only in the transcriptome data. Manual curation confirmed their presence in at least one of the replicates of the corresponding exome sequencing results where the coverage is highly dependent on the efficiency of the enrichment protocol. One of these SNS, *PRDM10*^S1050F^, was found to be immunogenic. Especially splicing and dynamic gene expression ranges complicate robust variant detection in RNA-seq data as compared with variant detection in exome or genome sequencing data [28]. Further development of mapping tools and variant callers specifically for RNA-seq data will enable more reliable detection of expressed mutations. Based on our experience in the Ma-Mel-86 model we would recommend to include *a priori* transcriptome sequencing for the identification of immunogenic mutations.

Ma-Mel-86a cells, carrying all HLA class I alleles on their cell surface, and Ma-Mel-86c cells, characterized by a haplotype loss, harbored 145 of the 181 somatic non-synonymous SNS (Figure 1) and nine of the ten somatic non-synonymous indels. Eighty of these 145 SNS and six of these nine indels were predicted to encode HLA class I-binding peptides, which were finally subjected to immunogenicity testing. As effectors for peptide testing we chose autologous CD8^+^ MLTC responder populations. Stimulators were Ma-Mel-86a and −86c cells as well as Ma-Mel-86c cells reconstituted with the HLA class I alleles of the lost haplotype (Table 2). These HLA transfectants were generated to detect also T cell responses against mutated peptides, named 86^NP^, that were encoded by somatic non-synonymous mutations present in Ma-Mel-86c, but not in Ma-Mel-86a, and that were predicted to bind solely to allele products of the lost HLA class I haplotype of Ma-Mel-86c. In the blood samples tested, no responses were detected against any of the 86c^NP^ peptides. All mutated neoantigens were restricted by HLA alleles that were present in Ma-Mel-86a and absent in Ma-Mel-86c. Therefore, HLA class I reconstitution of Ma-Mel-86c did not lead to the detection of additional immunogenic SNS. None of the indel-derived peptide candidates were immunogenic. Thirty-six of 181 SNS were only found in Ma-Mel-86b and −86f (Figure 1) and one expressed insertion was specific for Ma-Mel-86b. These were not included in immunogenicity testing.

Four of the 80 tested SNS (5%) were found to encode immunogenic peptides. Others reported similar success rates [8, 19]. Limitations of HLA-binding prediction algorithms, in particular uncertainties with respect to HLA-C binding [29] and a bias favoring peptides with predicted high binding affinity [30], are considered to lead to an underestimation of immunogenic SNS. Testing the actual peptide-binding affinity to the patients’ HLA alleles *in vitro* beforehand might lead to superior detection rates [17]. This is in line with a recent HLA peptidomics study that detected a mutated peptide presented on tumor cell surface that was predicted to be a weak binder [31]. The screening of tandem minigene libraries to bypass peptide prediction has not been shown to improve the detection of mutated neoantigens so far [18].

Only one of the four neoantigens, HERPUD1^mut^, had been identified via cDNA expression screening performed as described [15]. In general, the failure to identify the other three could be due to several reasons. cDNAs encoding the immunogenic mutations might have been underrepresented in the screened cDNA libraries due to low-level expression of the affected genes, due to a growth disadvantage of bacteria upon transformation with the antigen-encoding plasmids or due to a 3′-bias in cDNA libraries generated with oligo-dT primers. Furthermore, neoantigen-specific T cells within the T cell populations used for library screening might not have been sufficiently enriched to detect their signal.

Ma-Mel-86 neoantigens were identified with MLTC responder lymphocytes stimulated with autologous tumor cells for several weeks prior to testing of synthetic peptides. This *a priori* implied that they were able to induce T cell expansion when they were naturally expressed in autologous tumor cells. Intracellular processing and specific recognition of all four mutated neoantigens was unequivocally demonstrated by testing of cells transfected with mutated and wild-type cDNA with tumor-reactive clonal T cells (Figure 3).

In a retrospective analysis of 35 known T cell-defined mutated human tumor antigens, Fritsch *et al*. found that the mutations were predominantly located at non-anchor positions [32]. In contrast, profiling of mutational neoepitopes in mouse tumor models revealed that immunoprotective peptides preferentially carried mutations creating new anchor residues and were even characterized by MHC class I affinities considerably above the 500 nM threshold [30]. From the identified mutated Ma-Mel-86 neoantigens, only the serine-to-phenylalanine exchange at position 1050 of PRDM10^mut^_1042–1050_ had generated an anchor residue known to enable or considerably improve peptide binding to HLA-A*24:02 [33]. Whereas HERPUD1^mut^ and MMS22L^mut^ cannot be categorized with sufficient certainty, we assumed for INSIG1^mut^_234–241_ that the amino acid exchange at position 238 involved a T cell receptor-facing residue [34].

Among the four immunogenic mutations, only the mutation in *MMS22L*, which was located within a conserved region of the gene [35], was present in all four melanoma cell lines (Figure 1 and Table 3). *MMS22L* has been described as an oncogene in lung and esophageal cancer [36]. Therefore, *MMS22L*^mut^ in Ma-Mel-86 might carry a driver mutation, whereas the other three immunogenic neoantigens are more likely attributable to passenger mutations.

With an *ex vivo* ELISpot assay no reactivity against any of the four neoantigens was detectable in PBMCs cryopreserved in 08/2004. Deep sequencing of TCRβ CDR3 regions allowed to detect T cells against all four mutated neoantigens in PBMCs from 08/2004 at frequencies below 4 in 10^5^. Low frequency and transient responses against mutated neoantigens have also been observed by others [19]. Notably, only the clonotype against MMS22L^mut^ was detectable in PBMCs from 05/2002. T cells against the stably expressed neoantigen MMS22L^mut^ obviously contributed to the anti-tumor response already during the early disease phase.

From all Ma-Mel-86 melanoma cell lines only Ma-Mel-86a expressed HLA-A*24:02 and -B*15:01 that restricted the recognition of the four mutated neoantigens. This explained why only Ma-Mel-86a cells were recognized by neoantigen-specific T cells. Ma-Mel-86a had been derived from the earliest LN metastasis. The resistance of melanoma cell lines derived from later occurring metastases to neoantigen-reactive T cells supports the notion that T cells directed against tumor-specific mutations are strongly involved in immunoediting [37]. Accordingly, Zhao *et al*. [20] observed distinct T cell repertoires stimulated by Ma-Mel-86a and Ma-Mel-86c. In analogy to observations e.g. in renal cell cancer [38], we detected a considerable heterogeneity for somatic non-synonymous SNS across the metastases-derived tumor cell lines and phylogenetic reconstruction revealed a branched and not sequential evolution [20]. This indicated that tumor heterogeneity existed early on, and that the interaction with the patient's immune system led to the outgrowth of pre-existing immunoresistant tumor cell variants. Sequential immune escape was also observed in other patient models [39, 40] as discussed in [20].

T cell-oriented active immunotherapeutic interventions aim at augmenting inefficient T cell responses and broadening the anti-tumor T cell repertoire by recruiting also responses against subdominant epitopes. These objectives can be achieved both by checkpoint inhibition and by vaccination with neoantigens [7, 41]. Clinical testing of combined modalities is an obvious next step to go for [42], which is encouraged by animal studies [43]. Using mutated neoantigens for vaccination is supported by their strict tumor specificity, the lack of central immune tolerance, evidence that neoantigen burden of tumors correlates with the clinical effects of checkpoint inhibitors [2–5], and the increasing ease, with which they can be identified in individual patients.

In the light of a branched evolution, it appears unlikely that immunotherapeutic interventions relying on the presence of an intact HLA class I presentation will be sufficiently effective to prevent the outgrowth of HLA loss tumor cell variants even when applied in early disease. Increasing effectivity of treatments leading to longer survival in the presence of metastatic disease might even cause an increasing incidence of metastatic lesions exhibiting HLA class I loss [44]. But other, HLA class I-independent effector mechanisms might step in, such as CD4^+^ tumor-reactive T cells with direct effects on HLA class II-positive and indirect effects on HLA class II-negative tumors [45–47] as well as natural killer cells [48]. Immunotherapies involving diverse mechanisms of tumor recognition will therefore have best chances to induce sustained effects.

## MATERIALS AND METHODS

### Patient material

The human melanoma model Ma-Mel-86 (Supplementary Figure 1) consisted of the four melanoma cell lines Ma-Mel-86a, −86b, −86c and −86f, PBMCs from three blood donations and the autologous lymphoblastoid cell line Ma-Mel-86-EBV-B. Ma-Mel-86a cells expressed HLA-A*01:01, -A*24:02, -B*08:01, -B*15:01, -C*03:03 and -C*07:01, while Ma-Mel-86b and −86f were lacking HLA class I expression. Ma-Mel-86c expressed only one HLA class I haplotype with HLA-A*01:01, -B*08:01 and -C*07:01 [20].

### Cell lines

All patient-derived cell lines, COS-7 and 293T cells were maintained in standard culture medium (RPMI 1640 with L-glutamine supplemented with 10% FCS and 1% penicillin/streptomycin; Sigma, Steinheim, Germany).

### Preparation of genomic DNA and total RNA samples

Cryopreserved samples of all four melanoma cell lines with passage numbers >20 were thawed and seeded in culture flasks in standard culture medium to obtain a confluency of 70–80% within seven days (Supplementary Figure 2A). Medium was exchanged every 2–3 days. On day 7, the cells were harvested after trypsinization (Sigma, Steinheim, Germany). For each line, part of the cells was reseeded in culture for another week. From the other part genomic DNA (gDNA) or total RNA (first replicates) were isolated with QIAGEN kits QIAamp DNA Mini and RNeasy Mini (Hilden, Germany) according to the manufacturer's instructions. The reseeded cells were harvested on day 14 and applied to gDNA and RNA isolation as described above (second replicates). For the third RNA replicate, further cryopreserved samples of all four melanoma cell lines were thawed and the procedure was repeated until day 7. In addition, total RNA and gDNA were extracted from continuous cultures of the germline control line Ma-Mel-86-EBV-B.

### Construction of sequencing libraries and sequencing

Exome and transcriptome sequencing libraries were constructed by GENterprise Genomics (Mainz, Germany) using TruSeq™ DNA and RNA sample preparation kits (Illumina, Eindhoven, The Netherlands). Exome enrichment in the gDNA library was performed with the TruSeq™ Exome Enrichment Kit (Illumina, Eindhoven, The Netherlands). Average library sizes were 465 bp for exomes and 320 bp for transcriptomes. Exome (duplicates) and transcriptome (triplicates) libraries were subjected to high throughput sequencing on the Illumina HiSeq 2500 platform (NGS Unit, Johannes Gutenberg University, Mainz) generating 100 bp paired-end reads.

### Mapping and variant calling

Adapter sequences in the raw sequencing reads (100 bp, paired-end) were removed and quality trimming was performed using Trimmomatic v.0.22 [49] with trimming steps ILLUMINACLIP (allowed seed mismatches: 2, palindrome threshold: 40, simple threshold: 15), LEADING and TRAILING (minimal required quality: 13) and SLIDINGWINDOW (window size: 4, minimal required quality: 15). Trimmed reads shorter than 30 bp were rejected (MINLEN). Quality filter-passing exomic and transcriptomic reads were aligned to the human reference genome hg19 (UCSC, February 2009) using Bowtie2 version 2.0.0-beta6 [50] and TopHat version v2.0.3 [51], respectively. Variant calling in the exomes and transcriptomes was performed using SAMtools varFilter version 0.1.18 [52]. The tool snpEff version 3.1m [53] was used for the annotation of the detected mutations.

### Determination of expressed somatic non-synonymous SNS and indels

Somatic mutations were determined by subtracting variants found in the exome or transcriptome sequencing data of germline control Ma-Mel-86-EBV-B. Replicates were compared and variants lacking validation by at least a second replicate of the same sequencing procedure were excluded. Subtractions and intersections were generated with VCFtools version v0.1.9.0 [54]. Non-synonymous mutations in protein-coding regions were identified via snpEff annotation. The Integrative Genome Viewer (IGV) [22] was used for manual inspection of the detected SNS that were selected if they were present in at least two of three transcriptome replicates and at least one of two exome replicates. The context sequences of all somatic indels that were present in both exome replicates of at least one melanoma cell line were extracted and used to quantify indel-spanning RNA-seq reads using kallisto (version 0.43.0, [55]). Indels that were confirmed by at least 20 reads were considered as expressed.

### Prediction of HLA class I binding probability and synthetic peptides

For the prediction of the HLA binding capacity of peptides (octa-, nona- and decamers) containing the defined SNS, the relevant portions of the mutated protein sequences were extracted in an automated manner using own Perl scripts. These fragments were centered at the mutated residue and had a length of 15, 17 and 19 amino acids, respectively. For indels causing a frameshift, the protein region considered for peptide prediction comprised seven, eight or nine amino acids upstream of the mutation and the novel amino acid stretch until the first downstream stop codon. For in-frame indels, seven, eight or nine wild-type amino acids up- and downstream of the indel were considered for prediction. Novel amino acid stretches within the predicted peptides are underlined in Supplementary Table 1.

HLA binding prediction was performed with the algorithms of NetMHC 3.4 [23] and the IEDB MHC class I prediction tool [24]. Peptides with a predicted binding affinity of ≤ 500 nM (NetMHC) and a percentile rank of ≤ 10 (IEDB) were chosen for further analysis.

Synthetic peptides (see Supplementary Table 1) were synthesized by Peptide2.0 (Chantilly, VA, USA) at a purity of 20–80%. Lyophilized peptides were reconstituted in DMSO (40 mg/ml, Merck, Darmstadt, Germany) and diluted in PBS (200 μg/ml, Sigma, Steinheim, Germany).

### MLTCs and T cell clones

MLTCs were generated as described before [15]. MLTCs and clonal T cells were cultured in AIM-V medium (Gibco, Berlin, Germany) supplemented with 10% human serum. MLTC-derived CD8^+^ T cell clones were isolated via limiting dilution of the respective CD8^+^ MLTC responder populations and expanded in the presence of 250 U/ml rhIL-2 by weekly restimulation with 3 × 10^3^ Ma-Mel-86a or HLA-reconstituted Ma-Mel-86c cells per well and 5 × 10^4^ allogeneic lymphoblastoid cells per well as feeder cells that both were irradiated with 100 Gy. Expanding T cell clones were transferred into 48- or 24-well culture plates and restimulated with 5 × 10^4^ or 1 × 10^5^ stimulator cells and 1 × 10^5^ or 2 × 10^5^ feeder cells per well. Additionally, T cell clones 1A/1003, 3A/115 and 16C/114 were non-specifically expanded over 14 days with the anti-CD3 monoclonal antibody OKT3 (30 ng/ml), 250 U/ml rhIL-2 (Novartis Pharma, Nürnberg, Germany), 2.5 ng/ml rhIL-15 (Miltenyi Biotech, Bergisch Gladbach, Germany), irradiated PBMCs pooled from at least three healthy donors (2.5 × 10^7^ PBMCs per 0.1–0.2 × 10^5^ T cells) and irradiated allogeneic lymphoblastoid cells (5 × 10^6^ cells per 0.1–0.2 × 10^5^ T cells).

### Cloning of HLA class I alleles and generation of HLA transfectants

cDNAs encoding HLA-A*01:01, -A*24:02, -B*08:01, -B*15:01, -C*03:03 or -C*07:01 were cloned by RT-PCR in pcDNA3.1 as described [56]. Three stable transfectants of Ma-Mel-86c were generated by electroporation (140 V, 25 ms) with plasmids encoding HLA-A*24:02, HLA-B*15:01 and HLA-C*03:03, respectively. For each of the patient's HLA class I alleles, 293T transfectants were generated via electroporation (110 V, 25 ms) of 3 × 10^6^293T cells with 10 μg transgene-encoding plasmid-DNA in 100 μl OptiMEM (Sigma, Steinheim, Germany). On day 1 after electroporation, 10,000 transfected cells per allele and per well were pooled and used as peptide-presenting cells in IFNγ ELISpot assays.

### Cloning of full length and fragmented cDNAs encoding mutated neoantigens

*HERPUD1*- (isoform 1, NM_014685) and *INSIG1*-encoding (isoform 1, NM_005542) full-length and truncated cDNAs (mutated and wild-type) as well as truncated *MMS22L*-cDNAs (NM_198468) ending with codon 438 were cloned into pcDNA™3.1/V5-His TOPO^®^ using the pcDNA3.1/V5-His TOPO TA Cloning Kit^®^ (Invitrogen, Leek, The Netherlands). Full-length and truncated *PRDM10*-cDNAs (isoform 2, NM_199437) were inserted into the Gateway-compatible destination vector pcDNA3.1 DEST #6 using the Gateway system (Invitrogen, Leek, The Netherlands). 3′-fragmentation of the cDNAs was performed by PCR. Specific sense primers (containing the natural ATG start codon) were used together with different antisense primers that contained artificial stop codons for the amplification and cloning of different 3′-truncated fragments. For the 3′-fragments of *PRDM10*, an internal open reading frame starting with codon 845 encoding methionine was used. All primers used for cloning are listed in Supplementary Table 2. Correct orientation of the fragments was verified by restriction digestion as well as sequencing. In the recognition assays, 20,000 COS-7 or 293T cells per well were cotransfected on MultiScreen filter plates (Millipore, Eschborn, Germany) with the antigen-encoding cDNAs (300 ng/well) and cDNA encoding one of the patient's HLA class I alleles (100 ng/well) using 0.4–0.5 μl Lipofectamine^®^2000 (Invitrogen, Leek, The Netherlands) per reaction. T cell recognition of the cDNA recipient cells was assayed after 24 h in IFNγ ELISpot assays.

### IFNγ ELISpot assays

IFNγ ELISpot assays were performed as described [15] with 1–5 × 10^4^ effector cells per well in RPMI 1640 (Sigma, Steinheim, Germany) with 10% FCS (Sigma, Steinheim, Germany) and 250 U/ml rhIL-2 (Novartis Pharma, Nürnberg, Germany), if not otherwise specified. Peptide recognition was assayed with 10 μg/ml peptides in the presence (MLTC 3A) or absence (all other MLTCs) of 6 × 10^4^ 293T cells per well that had been electroporated with the patient's HLA class I alleles one day before. For the *ex vivo* assay [25], the recognized peptides encoded by *HERPUD1*^mut^, *INSIG1*^mut^, *MMS22L*^mut^ and *PRDM10*^mut^, respectively, were pooled and the reactivity of the patient's PBMCs (5 × 10^5^ cells per well) against the four pools was analyzed in serum-free AIM-V (Gibco, Berlin, Germany) without rhIL-2. The final concentration for each peptide was adjusted to 1 μg/ml. PBMCs incubated with phytohaemagglutinin E (Biochrom, Berlin, Germany) served as positive controls. All samples were tested in duplicates or triplicates. Readout was performed on an ImmunoSpot^®^ Analyzer (CTL Europe, Bonn, Germany). For peptide screening in ELISpot assays, the threshold for positivity was set to mean background plus three SD. Response definition in *ex vivo* ELISpot assays followed the first empirical rule given in [26], i.e. five spots per 10^5^ PBMCs with at least twofold increase of counts over background.

### Determination of the TCR Vβ sequences and TCR Vβ repertoire analysis by deep sequencing

As a first approximation, the TCR Vβ usage of neoantigen-specific T cell clones was analyzed with the IOTest Beta Mark TCR Vβ repertoire kit (Beckman Coulter, Krefeld, Germany). Their TCR Vβ sequences were determined by TCR Vβ-RT-PCR using primers published in [57] and Sanger sequencing of PCR products (Supplementary Table 3). TCR Vβ deep sequencing was performed on PBMCs collected in 05/2002 and in 08/2004 (Supplementary Figure 1) using the ImmunoSEQ assay platform (Adaptive Biotechnologies, Seattle) and the frequencies of the neoantigen-specific TCRβ clonotypes were calculated (Supplementary Table 3).

### mRNA expression analysis

Raw read counts determined with R/Bioconductor package GenomicRanges version 1.10.7 [58] were normalized to library size factors with DESeq version 1.10.1 [59] and to the length of the open reading frame of the respective genes. Circos version 0.67-pre5 [60] was used for the visualization of the expression levels of the SNS-affected genes in Figure 1.

### Data access

Raw sequencing data of the four melanoma cell lines and the EBV-B cells have been submitted to the Sequence Read Archive (SRA) under SRA project ID SRP068803. Mutated immunogenic cDNA sequences are accessible at NCBI GenBank (*INSIG1*^mut^: KU720376, *HERPUD1*^mut^: KU720377, *PRDM10*^mut^: KU720378 and *MMS22L*^mut^: KU720379).

## SUPPLEMENTARY MATERIALS FIGURES AND TABLES





## References

[1] Kreamer KM (2014). Immune Checkpoint Blockade: A New Paradigm in Treating Advanced Cancer. J Adv Pract Oncol.

[2] Champiat S, Ferté C, Lebel-Binay S, Eggermont A, Soria JC (2014). Exomics and immunogenics: Bridging mutational load and immune checkpoints efficacy. Oncoimmunology.

[3] Snyder A, Makarov V, Merghoub T, Yuan J, Zaretsky JM, Desrichard A, Walsh LA, Postow MA, Wong P, Ho TS, Hollmann TJ, Bruggeman C, Kannan K (2014). Genetic basis for clinical response to CTLA-4 blockade in melanoma. N Engl J Med.

[4] Rizvi NA, Hellmann MD, Snyder A, Kvistborg P, Makarov V, Havel JJ, Lee W, Yuan J, Wong P, Ho TS, Miller ML, Rekhtman N, Moreira AL (2015). Mutational landscape determines sensitivity to PD-1 blockade in non–small cell lung cancer. Science.

[5] Le JN, Uram DT, Wang H, Bartlett BR, Kemberling H, Eyring AD, Skora AD, Luber BS, Azad NS, Laheru D, Biedrzycki B, Donehower RC, Zaheer A (2015). PD-1 Blockade in Tumors with Mismatch-Repair Deficiency. N Engl J Med.

[6] van Rooij N, van Buuren MM, Philips D, Velds A, Toebes M, Heemskerk B, van Dijk LJA, Behjati S, Hilkmann H, El Atmioui D, Nieuwland M, Stratton MR, Kerkhoven RM (2013). Tumor exome analysis reveals neoantigen-specific T-cell reactivity in an ipilimumab-responsive melanoma. J Clin Oncol.

[7] Cha E, Klinger M, Hou Y, Cummings C, Ribas A, Faham M, Fong L (2014). Improved survival with T cell clonotype stability after anti-CTLA-4 treatment in cancer patients. Sci Transl Med.

[8] Rajasagi M, Shukla SA, Fritsch EF, Keskin DB, DeLuca D, Carmona E, Zhang W, Sougnez C, Cibulskis K, Sidney J, Stevenson K, Ritz J, Neuberg D (2014). Systematic identification of personal tumor-specific neoantigens in chronic lymphocytic leukemia. Blood.

[9] Hanson HL, Donermeyer DL, Ikeda H, White JM, Shankaran V, Old LJ, Shiku H, Schreiber RD, Allen PM (2000). Eradication of established tumors by CD8+ T cell adoptive immunotherapy. Immunity.

[10] Castle JC, Kreiter S, Diekmann J, Löwer M, van de Roemer N, de Graaf J, Selmi A, Diken M, Boegel S, Paret C, Koslowski M, Kuhn AN, Britten CM (2012). Exploiting the mutanome for tumor vaccination. Cancer Res.

[11] Lu YC, Yao X, Li YF, El-Gamil M, Dudley ME, Yang JC, Almeida JR, Douek DC, Samuels Y, Rosenberg SA, Robbins PF (2013). Mutated PPP1R3B is recognized by T cells used to treat a melanoma patient who experienced a durable complete tumor regression. J Immunol.

[12] Tran E, Turcotte S, Gros A, Robbins PF, Lu Y-C, Dudley ME, Wunderlich JR, Somerville RP, Hogan K, Hinrichs CS, Parkhurst MR, Yang JC, Rosenberg SA (2014). Cancer immunotherapy based on mutation-specific CD4+ T cells in a patient with epithelial cancer. Science.

[13] Baurain JF, Colau D, van Baren N, Landry C, Martelange V, Vikkula M, Boon T, Coulie PG (2000). High frequency of autologous anti-melanoma CTL directed against an antigen generated by a point mutation in a new helicase gene. J Immunol.

[14] Karanikas V, Colau D, Baurain JF, Chiari R, Thonnard J, Gutierrez-Roelens I, Goffinet C, Van Schaftingen EV, Weynants P, Boon T, Coulie PG (2001). High frequency of cytolytic T lymphocytes directed against a tumor-specific mutated antigen detectable with HLA tetramers in the blood of a lung carcinoma patient with long survival. Cancer Res.

[15] Lennerz V, Fatho M, Gentilini C, Frye RA, Lifke A, Ferel D, Wölfel C, Huber C, Wölfel T (2005). The response of autologous T cells to a human melanoma is dominated by mutated neoantigens. Proc Natl Acad Sci U S A.

[16] Galon J, Costes A, Sanchez-Cabo F, Kirilovsky A, Mlecnik B, Lagorce-Pagès C, Tosolini M, Camus M, Berger A, Wind P, Zinzindohoué F, Bruneval P, Cugnenc PH (2006). Type, density, and location of immune cells within human colorectal tumors predict clinical outcome. Science.

[17] Robbins PF, Lu Y-C, El-Gamil M, Li YF, Gross C, Gartner J, Lin JC, Teer JK, Cliften P, Tycksen E, Samuels Y, Rosenberg SA (2013). Mining exomic sequencing data to identify mutated antigens recognized by adoptively transferred tumor-reactive T cells. Nat Med.

[18] Lu Y-C, Yao X, Crystal JS, Li YF, El-Gamil M, Gross C, Davis L, Dudley ME, Yang JC, Samuels Y, Rosenberg SA, Robbins PF (2014). Efficient identification of mutated cancer antigens recognized by T cells associated with durable tumor regressions. Clin Cancer Res.

[19] Wick DA, Webb JR, Nielsen JS, Martin SD, Kroeger DR, Milne K, Castellarin M, Twumasi-Boateng K, Watson PH, Holt RA, Nelson BH (2014). Surveillance of the tumor mutanome by T cells during progression from primary to recurrent ovarian cancer. Clin Cancer Res.

[20] Zhao F, Sucker A, Horn S, Heeke C, Bielefeld N, Schrörs B, Bicker A, Lindemann M, Roesch A, Gaudernack G, Stiller M, Becker JC, Lennerz V (2016). Melanoma Lesions Independently Acquire T-cell Resistance during Metastatic Latency. Cancer Res.

[21] Hill VK, Gartner JJ, Samuels Y, Goldstein AM (2013). The genetics of melanoma: recent advances. Annu Rev Genomics Hum Genet.

[22] Thorvaldsdóttir H, Robinson JT, Mesirov JP (2013). Integrative Genomics Viewer (IGV): high-performance genomics data visualization and exploration. Brief Bioinform.

[23] Lundegaard C, Lamberth K, Harndahl M, Buus S, Lund O, Nielsen M (2008). NetMHC-3.0: accurate web accessible predictions of human, mouse and monkey MHC class I affinities for peptides of length 8-11. Nucleic Acids Res.

[24] Zhang Q, Wang P, Kim Y, Haste-Andersen P, Beaver J, Bourne PE, Bui H-H, Buus S, Frankild S, Greenbaum J, Lund O, Lundegaard C, Nielsen M (2008). Immune epitope database analysis resource (IEDB-AR). Nucleic Acids Res.

[25] Britten CM, Gouttefangeas C, Welters MJP, Pawelec G, Koch S, Ottensmeier C, Mander A, Walter S, Paschen A, Müller-Berghaus J, Haas I, Mackensen A, Køllgaard T (2008). The CIMT-monitoring panel: a two-step approach to harmonize the enumeration of antigen-specific CD8+ T lymphocytes by structural and functional assays. Cancer Immunol Immunother.

[26] Moodie Z, Price L, Gouttefangeas C, Mander A, Janetzki S, Löwer M, Welters MJP, Ottensmeier C, van der Burg SH, Britten CM (2010). Response definition criteria for ELISPOT assays revisited. Cancer Immunol Immunother.

[27] Flockhart RJ, Webster DE, Qu K, Mascarenhas N, Kovalski J, Kretz M, Khavari PA (2012). BRAFV600E remodels the melanocyte transcriptome and induces BANCR to regulate melanoma cell migration. Genome Res.

[28] Tang X, Baheti S, Shameer K, Thompson KJ, Wills Q, Niu N, Holcomb IN, Boutet SC, Ramakrishnan R, Kachergus JM, Kocher J-PA, Weinshilboum RM, Wang L (2014). The eSNV-detect: a computational system to identify expressed single nucleotide variants from transcriptome sequencing data. Nucleic Acids Res.

[29] Walshe VA, Hattotuwagama CK, Doytchinova IA, Wong M, Macdonald IK, Mulder A, Claas FHJ, Pellegrino P, Turner J, Williams I, Turnbull EL, Borrow P, Flower DR (2009). Integrating in silico and *in vitro* analysis of peptide binding affinity to HLA-Cw*0102: a bioinformatic approach to the prediction of new epitopes. PloS One.

[30] Duan F, Duitama J, Al Seesi S, Ayres CM, Corcelli SA, Pawashe AP, Blanchard T, McMahon D, Sidney J, Sette A, Baker BM, Mandoiu II, Srivastava PK (2014). Genomic and bioinformatic profiling of mutational neoepitopes reveals new rules to predict anticancer immunogenicity. J Exp Med.

[31] Kalaora S, Barnea E, Merhavi-Shoham E, Qutob N, Teer JK, Shimony N, Schachter J, Rosenberg SA, Besser MJ, Admon A, Samuels Y (2016). Use of HLA peptidomics and whole exome sequencing to identify human immunogenic neo-antigens. Oncotarget.

[32] Fritsch EF, Rajasagi M, Ott PA, Brusic V, Hacohen N, Wu CJ (2014). HLA-Binding Properties of Tumor Neoepitopes in Humans. Cancer Immunol Res.

[33] Sidney J, Southwood S, Sette A (2005). Classification of A1- and A24-supertype molecules by analysis of their MHC-peptide binding repertoires. Immunogenetics.

[34] Rudolph MG, Stanfield RL, Wilson IA (2006). How TCRs bind MHCs, peptides, and coreceptors. Annu Rev Immunol.

[35] Duro E, Lundin C, Ask K, Sanchez-Pulido L, MacArtney TJ, Toth R, Ponting CP, Groth A, Helleday T, Rouse J (2010). Identification of the MMS22L-TONSL complex that promotes homologous recombination. Mol Cell.

[36] Nguyen M-H, Ueda K, Nakamura Y, Daigo Y (2012). Identification of a novel oncogene, MMS22L, involved in lung and esophageal carcinogenesis. Int J Oncol.

[37] Matsushita H, Vesely MD, Koboldt DC, Rickert CG, Uppaluri R, Magrini VJ, Arthur CD, White JM, Chen Y-S, Shea LK, Hundal J, Wendl MC, Demeter R (2012). Cancer exome analysis reveals a T-cell-dependent mechanism of cancer immunoediting. Nature.

[38] Gerlinger M, Horswell S, Larkin J, Rowan AJ, Salm MP, Varela I, Fisher R, McGranahan N, Matthews N, Santos CR, Martinez P, Phillimore B, Begum S (2014). Genomic architecture and evolution of clear cell renal cell carcinomas defined by multiregion sequencing. Nat Genet.

[39] Coulie PG, Ikeda H, Baurain JF, Chiari R (1999). Antitumor immunity at work in a melanoma patient. Adv Cancer Res.

[40] Yamshchikov GV, Mullins DW, Chang C-C, Ogino T, Thompson L, Presley J, Galavotti H, Aquila W, Deacon D, Ross W, Patterson JW, Engelhard VH, Ferrone S (2005). Sequential immune escape and shifting of T cell responses in a long-term survivor of melanoma. J Immunol.

[41] Carreno BM, Magrini V, Becker-Hapak M, Kaabinejadian S, Hundal J, Petti AA, Ly A, Lie W-R, Hildebrand WH, Mardis ER, Linette GP (2015). Cancer immunotherapy. A dendritic cell vaccine increases the breadth and diversity of melanoma neoantigen-specific T cells. Science.

[42] Trajanoski Z, Maccalli C, Mennonna D, Casorati G, Parmiani G, Dellabona P (2015). Somatically mutated tumor antigens in the quest for a more efficacious patient-oriented immunotherapy of cancer. Cancer Immunol Immunother.

[43] Bartkowiak T, Singh S, Yang G, Galvan G, Haria D, Ai M, Allison JP, Sastry KJ, Curran MA (2015). Unique potential of 4-1BB agonist antibody to promote durable regression of HPV+ tumors when combined with an E6/E7 peptide vaccine. Proc Natl Acad Sci USA.

[44] Tran E, Robbins PF, Lu Y-C, Prickett TD, Gartner JJ, Jia L, Pasetto A, Zheng Z, Ray S, Groh EM, Kriley IR, Rosenberg SA (2016). T-Cell Transfer Therapy Targeting Mutant KRAS in Cancer. N Engl J Med.

[45] Mumberg D, Monach PA, Wanderling S, Philip M, Toledano AY, Schreiber RD, Schreiber H (1999). CD4(+) T cells eliminate MHC class II-negative cancer cells *in vivo* by indirect effects of IFN-gamma. Proc Natl Acad Sci U S A.

[46] Schumacher T, Bunse L, Pusch S, Sahm F, Wiestler B, Quandt J, Menn O, Osswald M, Oezen I, Ott M, Keil M, Balß J, Rauschenbach K (2014). A vaccine targeting mutant IDH1 induces antitumour immunity. Nature.

[47] Kreiter S, Vormehr M, van de Roemer N, Diken M, Löwer M, Diekmann J, Boegel S, Schrörs B, Vascotto F, Castle JC, Tadmor AD, Schoenberger SP, Huber C (2015). Mutant MHC class II epitopes drive therapeutic immune responses to cancer. Nature.

[48] Pietra G, Vitale C, Pende D, Bertaina A, Moretta F, Falco M, Vacca P, Montaldo E, Cantoni C, Mingari MC, Moretta A, Locatelli F, Moretta L (2016). Human natural killer cells: news in the therapy of solid tumors and high-risk leukemias. Cancer Immunol Immunother.

[49] Bolger AM, Lohse M, Usadel B (2014). Trimmomatic: a flexible trimmer for Illumina sequence data. Bioinforma Oxf Engl.

[50] Langmead B, Salzberg SL (2012). Fast gapped-read alignment with Bowtie 2. Nat Methods.

[51] Trapnell C, Pachter L, Salzberg SL (2009). TopHat: discovering splice junctions with RNA-Seq. Bioinforma Oxf Engl.

[52] Li H, Handsaker B, Wysoker A, Fennell T, Ruan J, Homer N, Marth G, Abecasis G, Durbin R (2009). The Sequence Alignment/Map format and SAMtools. Bioinformatics.

[53] Cingolani P, Platts A, Wang LLL, Coon M, Nguyen T, Land SJ, Lu X, Ruden DM (2012). A program for annotating and predicting the effects of single nucleotide polymorphisms, SnpEff: SNPs in the genome of Drosophila melanogaster strain w1118; iso-2; iso-3. Fly (Austin).

[54] Danecek P, Auton A, Abecasis G, Albers C, Banks E, DePristo MA, Handsaker RE, Lunter G, Marth GT, Sherry ST, McVean G, Durbin R, 1000 Genomes Project Analysis Group (2011). The variant call format and VCFtools. Bioinforma Oxf Engl.

[55] Bray NL, Pimentel H, Melsted P, Pachter L (2016). Near-optimal probabilistic RNA-seq quantification. Nat Biotechnol.

[56] Ennis PD, Zemmour J, Salter RD, Parham P (1990). Rapid cloning of HLA-A, B cDNA by using the polymerase chain reaction: frequency and nature of errors produced in amplification. Proc Natl Acad Sci USA.

[57] Peggs K, Verfuerth S, Pizzey A, Ainsworth J, Moss P, Mackinnon S (2002). Characterization of human cytomegalovirus peptide-specific CD8(+) T-cell repertoire diversity following *in vitro* restimulation by antigen-pulsed dendritic cells. Blood.

[58] Lawrence M, Huber W, Pagès H, Aboyoun P, Carlson M, Gentleman R, Morgan MT, Carey VJ (2013). Software for computing and annotating genomic ranges. PLoS Comput Biol.

[59] Anders S, Huber W (2010). Differential expression analysis for sequence count data. Genome Biol.

[60] Krzywinski M, Schein J, Birol I, Connors J, Gascoyne R, Horsman D, Jones SJ, Marra MA (2009). Circos: an information aesthetic for comparative genomics. Genome Res.

