# Supplementary material for: HLA class I loss in metachronous metastases prevents continuous T cell recognition of mutated neoantigens in a human melanoma model
Source: Oncotarget. 2017 Mar 9;8(17):28312–27. doi: 10.18632/oncotarget.16048 (PMC5438652; doi:10.18632/oncotarget.16048)
Supplement: Supplementary file 1 [file oncotarget-08-28312-s001.pdf]

HLA class I loss in metachronous metastases prevents continuous T cell recognition of mutated neoantigens in a human melanoma model

Supplementary Materials

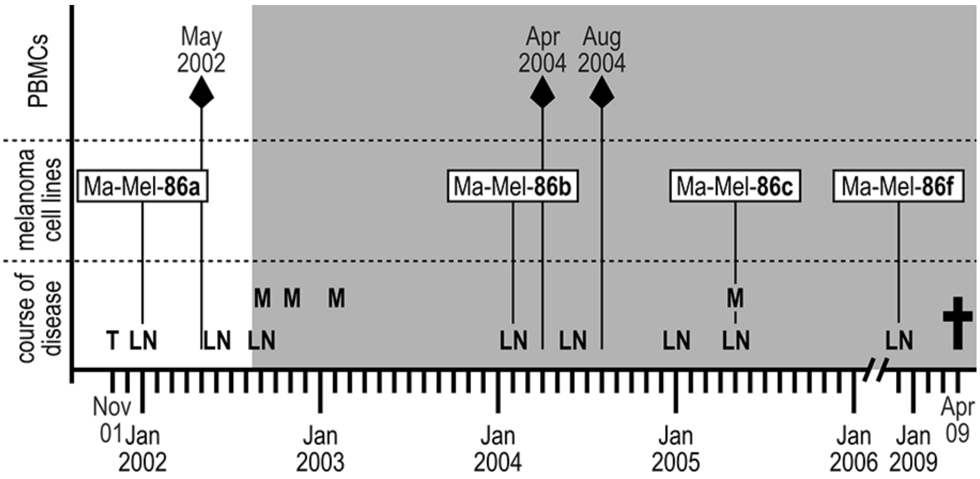

Supplementary Figure 1: Melanoma model Ma-Mel-86. The melanoma cell lines Ma-Mel-86a, -86b, -86c and -86f were established from four different, surgically removed lymph node metastases (LN) of patient Ma-Mel-86. Autologous PBMCs were available from blood donations performed in May 2002, April 2004 and August 2004. Despite her aggressive disease with several metastases in distant organs (M, comprising brain, breast and intestinal metastases), she survived almost eight years after the diagnosis of the primary tumor (T). Disease stage IV is indicated in gray.

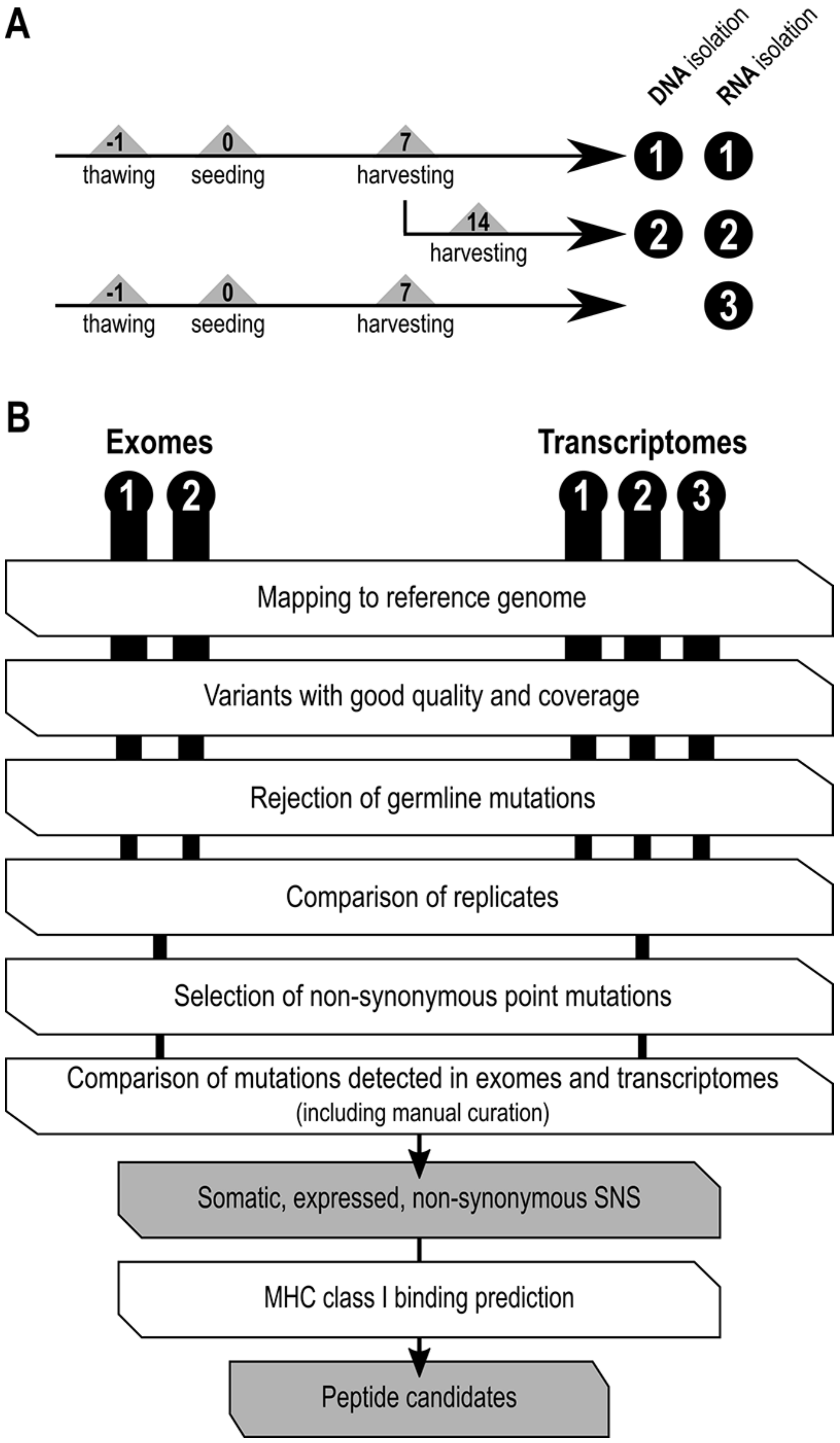

Supplementary Figure 2: Strategy for the localization of potentially immunogenic mutations in Ma-Mel-86. (**A**) For each Ma-Mel-86 cell line, two genomic DNA (black circles, replicates 1 and 2) and three total RNA preparations (replicates 1, 2 and 3) from two independent cultures (replicates 1 and 3) and two different time points (replicates 1 and 3: day 7; replicate 2: day 14) were generated for high throughput sequencing according to the depicted scheme starting with cryopreserved cells. Numbers in gray triangles indicate days. (**B**) The computational strategy to select for potentially immunogenic peptides carrying tumor-specific mutations was based on processing the generated replicate data (black circles) as long as possible in parallel. Somatic non-synonymous SNS detected in at least two of three transcriptomes and at least one of two exome replicates were selected.

Supplementary Table 1: Peptides selected for immunogenicity testing. See Supplementary_Table_1

Supplementary Table 2: Primers used for cloning of full length and 3′-fragmented neoantigen-encoding cDNAs

Construct	Cloned cDNA	Cloning technique	Primer (5′ → 3′) sense	Primer (5′ → 3′) antisense
INSIG1mut / INSIG1wt	full length	TOPO TA	**ATG**CCCAGATTGCACGACCAC	TCAATCACTATGGGGCTTTTCAGG
INSIG1mut_241(F)	3′-fragment	TOPO TA		**CTA**GAAATCTGGGAATGTATACTGATAG
INSIG1mut_240(D)	3′-fragment	TOPO TA		**CTA**ATCTGGGAATGTATACTGATAGAC
PRDM10mut / PRDM10wt	full length	Gateway	GGGGACAAGTTTGTACAAA AAAGCAGGCTTCACC**ATG**GATTC GAAAGATGAAAGCTC	GGGGACCACTTTGTACAAGAAAGCTGGGTC TCATGGTTTGGTGATATGCACTTC
PRDM10mut_1050(F)	internal ORF, 3′-fragment	Gateway	GGGGACAAGTTTGTACA AAAAAGCAGGCTTCAC C**ATG**GTCCAGCACATTCGAAAG	GGGGACCACTTTGTACAAGAAAGCTGGGT CCTAGAAATTCCAAGCACTGGGCAG
PRDM10mut_1049(N)	internal ORF, 3′-fragment	Gateway		GGGGACCACTTTGTACAAGAAAGCTGGGT C**CTA**ATTCCAAGCACTGGGCAG
MMS22Lmut_439(S)	3′-fragment	TOPO TA	**ATG**GAGAACTGTTCTGCTGC	**CTA**ACTGAAGAAACTATTCAGGTTCTTAC
MMS22Lmut_438(F)	3′-fragment	TOPO TA		**CTA**GAAGAAACTATTCAGGTTCTTACT
MMS22Lmut_437(F)	3′-fragment	TOPO TA		**CTA**GAAACTATTCAGGTTCTTACTATAATAT
MMS22Lwt_438(F)	3′-fragment	TOPO TA		**CTA**GAAGGAACTATTCAGGTTCTTACT
HERPUD1wt	full length	TOPO TA	ACCGCC**ATG**GGCGGCAGCC	TCAGTTTGCGATGGCTGG
HERPUD1mut_162(Y)	3′-fragment	TOPO TA		**CTA**GTAACTGGAGAAACCAGG
HERPUD1mut_161(S)	3′-fragment	TOPO TA		**CTA**ACTGGAGAAACCAGGACC
bold	initiation codon (**ATG**)			
bold and underlined	inserted stop codon (**CTA**)			
red	mutated nucleotide			

Supplementary Table 3: Detection of mutation-specific TCRβ clonotypes via deep sequencing of TCRβ CDR3 regions

T cell clones	HLA restriction	Target antigen	TRBV allele(*)	TRBD allele(*)	TRBJ allele(*)	CDR3 amino acid sequence	Total clonotypic reads (per 105 productive rearrangements) in PBMCs from	
							05/2002	08/2004
1A/39, 1A/108	HLA-B*15:01	HERPUD^{mut}	5-1*01	02-01*02	02-05*01	CASNQAGGPGETQYF	0	7 (1.6)
1A/1001, 1A/1003	HLA-A*24:02	PRDM10^{mut}	04-01*01	01-01*01	02-02*01	CASSEQGAGAGELFF	0	3 (0.7)
16C/26	HLA-A*24:02	INSIG1^{mut}	20-1*01-*05	02-01*02	02-01*01	CSAISRNDYNEQFF	0	1 (0.2)
3A/115	HLA-A*24:02	INSIG1^{mut}	20-1*01-*05	unresolved	01-04*01	CSARVRAGEKLFF	0	17 (3.8)
16C/106, 16C/92	HLA-A*24:02	MMS22L^{mut}	7-9*01	02-01*01	02-05*01	CASSFIGGVETQYF	20 (6.1)	6 (1.3)

(*) Nomenclature according to: http://www.imgt.org/IMGTScientificChart/Nomenclature/IMGTnomenclature.html
In total, 406,136 gene rearrangements (329,991 productive rearrangements) in blood sample 05/2002 and 547,390 gene rearrangements (445,925 productive rearrangements) in blood sample 08/2004 were analyzed.
