# Supplementary material for: HLA class I loss in metachronous metastases prevents continuous T cell recognition of mutated neoantigens in a human melanoma model
Source: Oncotarget. 2017 Mar 9;8(17):28312–27. doi: 10.18632/oncotarget.16048 (PMC5438652; doi:10.18632/oncotarget.16048)
Supplement: Supplementary file 2 [file oncotarget-08-28312-s002.pdf]

Supplementary Table 1: Peptides selected for immunogenicity testing. Gray shaded rows indicate peptides that were recognized by MLTC responders (Figure 2). Indel-derived peptides (novel amino acid stretches underlined) were tested separately and are thus not shown in Figure 2 (n/a: not applicable).

Peptide # in fig. 2	Peptide sequence	Gene symbol	Genomic position of mutation	Amino acid exchange	Mutation present and expressed in				IEDB [percentile rank]		NetMHC binding affinity [nM]		Predicted binding to HLA-...
					Ma-Mel-86a	Ma-Mel-86b	Ma-Mel-86c	Ma-Mel-86f	mutated peptide	wild-type peptide	mutated peptide	wild-type peptide	
1	AAWSTGPWSL	ADAMTS9	chr3:64579935	S1452L	-	+	+	+	6.842	60.526	28	16678	C*03:03
2	WSTGPWSL	ADAMTS9	chr3:64579935	S1452L	-	+	+	+	6.842	65.789	31	24579	C*03:03
3	FSYKSEMDSM	AHR	chr7:17379505	P686S	+	+	+	-	0.526	3.158	4	10	C*03:03
4	GISQEFSY	AHR	chr7:17379505	P686S	+	+	+	-	8.421	7.368	190	157	B*15:01
5	CRAPKVSQHV	AP1B1	chr22:29724860	E934K	+	+	+	+	2.105	3.158	62	96	C*07:01
6	KVSQHVYQAY	AP1B1	chr22:29724860	E904K	+	+	+	+	6.842	14.474	44	140	B*15:01
7	SLKCRAPKV	AP1B1	chr22:29724860	E904K	+	+	+	+	2.105	2.105	60	54	B*08:01
8	FPEPGSRVL	ARHGAP23	chr17:36622757	P278L	+	+	+	+	8.421	48.684	11	21058	C*03:03
9	SRVLPSRL	ARHGAP23	chr17:36622757	P278L	+	+	+	+	8.421	3.158	500	116	C*07:01
10	LWNMSDDKLF	ATF2	chr2:175994897	P24L	+	+	+	+	2.632	4.474	399	804	A*24:02
11	GVYDTPPM	BCAR1	chr16:75271082	E117G	-	-	+	-	4.211	6.316	13	27	C*03:03
12	SQYGQGVY	BCAR1	chr16:75271082	E117G	-	-	+	-	0.526	0.526	29	31	B*15:01
13	EASPGTADM	BEND5	chr1:49224814	E168A	+	+	+	+	5.263	7.895	32	51	C*03:03
14	LRDQELVQIV	BRWD1	chr21:40601347	K1006Q	+	+	+	+	4.737	8.421	175	433	C*07:01
15	VQIVGIRY	BRWD1	chr21:40601347	K1006Q	+	+	+	+	3.158	47.368	62	4874	B*15:01
16	PRSRYPQSSL	C11orf41	chr11:33689477	P1776L	+	-	-	-	7.368	63.158	334	18396	C*07:01
17	RYPQSSLSRL	C11orf41	chr11:33689477	P1776L	+	-	-	-	1.053	1.053	63	72	A*24:02
18	SRYPQSSL	C11orf41	chr11:33689477	P1776L	+	-	-	-	0.526	24.211	11	2466	C*07:01
19	SRYPQSSLS	C11orf41	chr11:33689477	P1776L	+	-	-	-	3.158	35.000	165	4014	C*07:01
20	SRYPQSSLSR	C11orf41	chr11:33689477	P1776L	+	-	-	-	8.421	14.211	410	1045	C*07:01
21	EMLLQALPL	C19orf6	chr19:1010809	P468L	+	+	+	+	5.789	11.579	144	536	B*08:01
22	MLLQALPL	C19orf6	chr19:1010809	P468L	+	+	+	+	8.947	7.368	60	40	C*03:03
23	LEAKSTSAIL	C1orf9	chr1:172539301	P287L	+	+	+	+	9.474	53.684	57	4043	C*03:03
24	NLEAKSTSA	C1orf9	chr1:172539301	P287L	+	+	+	+	5.789	6.316	196	195	B*08:01
25	ISANKEDL	C5	chr9:123725931	E1436K	-	-	+	-	8.421	10.526	52	79	C*03:03
26	RLHWFLLF	CCNL1	chr3:156867939	P265L	+	+	+	+	4.737	59.474	89	13726	B*15:01
27	TRLHWFLL	CCNL1	chr3:156867939	P265L	+	+	+	+	4.211	4.211	168	199	C*07:01
28	TRLHWFLLF	CCNL1	chr3:156867939	P265L	+	+	+	+	2.895	1.579	216	57	A*24:02
29	AHDPHSGHFV	CDK4	chr12:58145430	R24H	+	+	+	+	2.105	3.158	68	99	C*07:01
30	AQKEGTSQAL	CEP85	chr1:26601479	E607K	+	-	+	+	6.842	22.632	23	248	B*15:01
31	DLQEDVDACY	CNBP	chr3:128890122	E67D	+	-	-	-	10.000	12.895	370	586	B*15:01
32	LQEDDACY	CNBP	chr3:128890122	E66D	+	-	-	-	7.895	10.526	176	260	B*15:01
33	LQEDVDACY	CNBP	chr3:128890122	E67D	+	-	-	-	8.421	13.158	114	232	B*15:01
34	AAQHGHVDTL	DAPK1	chr9:90262251	R421Q	+	+	-	+	4.211	7.895	14	42	C*03:03
35	QHGHVDTL	DAPK1	chr9:90262251	R421Q	+	+	-	+	7.368	6.842	393	360	C*07:01
36	WAAQHGHV	DAPK1	chr9:90262251	R421Q	+	+	-	+	8.947	12.105	58	95	C*03:03
37	MPRANVCVV	DIP2A	chr21:47961805	G661R	+	-	+	+	2.632	12.632	65	1377	B*08:01

38	FQFSSVLNSH	ELK4	chr1:205585769	P401S	+	+	+	+	5.526	3.947	59	57	B*15:01
39	TLFQFSSVL	ELK4	chr1:205585769	P401S	+	+	+	+	7.895	26.316	97	1079	B*15:01
40	LLPTSSTSSL	EP400	chr12:132471111	P661L	+	+	+	+	9.211	31.842	88	1296	B*15:01
41	MAVPPSAPQQ	ETNK2	chr1:204120952	P10Q	+	+	+	+	10.000	22.105	64	344	C*03:03
42	QQRASFHL	ETNK2	chr1:204120952	P10Q	+	+	+	+	4.737	64.211	88	16364	B*15:01
43	WSAKKYPGDY	FAM213B	chr1:2520397	S192Y	+	+	+	+	1.842	1.316	350	273	A*01:01
44	GAVKVKEEPM	HDAC9	chr7:18706031	V475M	+	+	+	+	2.105	12.632	8	101	C*03:03
45	GHEDEEEPQL	HERC1	chr15:63958256	S2806L	+	-	+	-	5.263	57.368	208	11098	C*07:01
46	FSSYTPYGWL	HERPUD1	chr16:56973198	G136S	+	-	+	-	5.789	12.632	23	98	C*03:03
47	GLGPGFSSY	HERPUD1	chr16:56973198	G136S	+	-	+	-	2.105	6.842	31	58	B*15:01
48	SYTPYGWLQL	HERPUD1	chr16:56973198	G136S	+	-	+	-	2.105	4.474	233	819	A*24:02
49	DAVFPEPSL	HMHA1	chr19:1068501	G60E	-	-	+	+	5.526	3.947	20	8	C*03:03
50	ETLKRPTSL	HMHA1	chr19:1068501	G60E	-	-	+	+	1.579	5.789	48	310	B*08:01
51	FSVTDAVSF	HSHP1	chr13:31722560	P399S	-	+	+	+	4.211	3.684	55	41	B*15:01
52	IHSQISSI	INSIG1	chr7:155094465	P141S	+	+	+	-	1.579	3.158	41	125	C*07:01
53	IHSQISSIF	INSIG1	chr7:155094465	P141S	+	+	+	-	5.000	28.421	301	145	A*24:02
54	IHSQISSIFV	INSIG1	chr7:155094465	P141S	+	+	+	-	3.684	7.895	130	359	C*07:01
55	QYTFPDFLYI	INSIG1	chr7:155094465	S238F	+	+	+	-	2.632	2.632	306	261	A*24:02
56	SQISSIFVL	INSIG1	chr7:155094465	P141S	+	+	+	-	2.105	22.105	44	3179	B*15:01
57	TAAGIHSQI	INSIG1	chr7:155094465	P141S	+	+	+	-	4.737	5.000	23	26	C*03:03
58	VYNGVYQYTF	INSIG1	chr7:155094465	S238F	+	+	+	-	0.526	8.684	11	3353	A*24:02
59	VYQYTFPDF	INSIG1	chr7:155094465	S238F	+	+	+	-	0.526	1.316	15	46	A*24:02
60	VYQYTFPDFL	INSIG1	chr7:155094465	S238F	+	+	+	-	2.105	2.895	84	127	A*24:02
61	YQYTFPDF	INSIG1	chr7:155094465	S238F	+	+	+	-	0.526	0.526	30	26	B*15:01
62	YQYTFPDFLY	INSIG1	chr7:155094465	S238F	+	+	+	-	0.789	0.526	14	11	B*15:01
63	YTFPDFLY	INSIG1	chr7:155094465	S238F	+	+	+	-	4.211	6.316	74	128	B*15:01
64	LAWGGIFSF	IQCH	chr15:67664596	L301F	+	+	+	+	5.789	47.368	89	3979	B*15:01
65	LAWGGIFSFL	IQCH	chr15:67664596	L301F	+	+	+	+	0.526	0.526	4	4	C*03:03
66	SFLEHVEKF	IQCH	chr15:67664596	L301F	+	+	+	+	3.947	22.105	233	4107	A*24:02
67	SLAWGGIFSF	IQCH	chr15:67664596	L301F	+	+	+	+	2.368	22.632	42	930	B*15:01
68	KAFSYFKSF	IRS2	chr13:110435231	G1057D	-	+	+	+	2.105	6.316	39	97	B*15:01
69	ARRSGGALYI	KANK1	chr9:711797	E186A	+	+	+	+	2.632	2.632	76	70	C*07:01
70	RRSGGALYI	KANK1	chr9:711797	E186A	+	+	+	+	6.053	6.316	46	83	C*07:01
71	SRARRSGGAL	KANK1	chr9:711797	E186A	+	+	+	+	6.842	3.684	295	110	C*07:01
72	ELRKHLSL	LIMK2	chr22:31672891	N609S	+	+	+	+	5.000	5.000	288	360	B*08:01
73	TVLDQQQTL	LRPPRC	chr2:44128618	P1137L	+	-	+	+	9.474	52.368	13	25962	C*03:03
74	LSLNYLVL	LRRC58	chr3:120054735	P189L	+	+	+	+	7.368	38.947	42	1531	C*03:03
75	HRDVKSSNIL	MAP2K1	chr15:66774101	P193S	+	+	+	+	7.895	7.895	360	399	C*07:01
76	MHRDVKSSNI	MAP2K1	chr15:66774101	P193S	+	+	+	+	0.526	0.526	21	23	C*07:01
77	KFVEKCSLI	MCM5	chr22:35809972	P399L	+	+	+	+	4.211	9.211	296	1371	A*24:02
78	LLKFVEKCSL	MCM5	chr22:35809972	P399L	+	+	+	+	7.105	60.526	406	17796	B*08:01
79	AAGNSGSL	MDC1	chr6:30671772	P1730S	+	-	+	+	5.263	6.842	21	30	C*03:03
80	QRAAGNSGSL	MDC1	chr6:30671772	P1730S	+	-	+	+	7.368	7.895	343	356	C*07:01
81	RAAGNSGSL	MDC1	chr6:30671772	P1730S	+	-	+	+	1.316	2.895	3	7	C*03:03
82	RQRAAGNSG	MDC1	chr6:30671772	P1730S	+	-	+	+	4.737	3.684	44	38	B*15:01

83	VVRRPRAAF	METTL22	chr16:8722848	S132F	+	-	+	-	9.474	63.684	85	15364	B*15:01
84	EYYSKNLNSF	MMS22L	chr6:97679521	S437F	+	+	+	+	0.526	36.842	80	17551	A*24:02
85	FFSISWLPF	MMS22L	chr6:97679521	S437F	+	+	+	+	2.105	1.579	159	79	A*24:02
86	SFFSISWLPF	MMS22L	chr6:97679521	S437F	+	+	+	+	1.053	4.474	42	493	A*24:02
87	YSKNLNSFF	MMS22L	chr6:97679521	S437F	+	+	+	+	4.211	1.053	35	19	B*15:01
88	YYSKNLNSF	MMS22L	chr6:97679521	S437F	+	+	+	+	0.263	16.053	10	3866	A*24:02
89	YYSKNLNSFF	MMS22L	chr6:97679521	S437F	+	+	+	+	0.526	0.526	12	16	A*24:02
90	FIRKLVNPL	MRPL36	chr5:1799025	M9L	+	+	+	+	1.053	0.526	20	14	B*08:01
91	KLVNPLLY	MRPL36	chr5:1799025	M9L	+	+	+	+	10.000	4.737	249	83	B*15:01
92	LPLKFSKTL	MYO1E	chr15:59455358	N875K	+	-	+	-	3.158	3.684	106	124	B*08:01
93	ENKEKFEAF	OTUD4	chr4:146095829	R11K	+	+	+	+	3.684	3.684	138	137	B*08:01
94	HYLRENKEKF	OTUD4	chr4:146095829	R11K	+	+	+	+	2.105	2.368	302	349	A*24:02
95	FIDGKVLYV	PABPC5	chrX:90691364	S263F	+	+	+	+	2.632	6.579	458	3534	A*01:01
96	GPLTKTKSI	PARS2	chr1:55223925	G304S	+	+	+	+	5.263	23.684	115	2723	B*08:01
97	KSIEVGHTF	PARS2	chr1:55223925	G304S	+	+	+	+	2.105	13.684	39	282	B*15:01
98	KSIEVGHTFY	PARS2	chr1:55223925	G304S	+	+	+	+	8.421	16.579	49	412	B*15:01
99	SIEVGHTFY	PARS2	chr1:55223925	G304S	+	+	+	+	3.947	5.000	430	1115	A*01:01
100	IRINRCAI	PC	chr11:66633732	G371R	-	-	+	-	4.211	10.526	169	642	C*07:01
101	NIRINRCAI	PC	chr11:66633732	G371R	-	-	+	-	2.632	7.895	32	249	B*08:01
102	VAKKYHEVL	PDGFC	chr4:157688942	T302A	+	-	-	-	4.211	10.526	164	1081	B*08:01
103	ILFIPSGWIY	PHF2	chr9:96416840	T312I	+	-	+	-	2.368	6.053	32	51	B*15:01
104	ARKGRISV	PKP2	chr12:33049457	S70I	+	-	-	-	2.632	2.105	102	74	C*07:01
105	ISVGNGNL	PKP2	chr12:33049457	S70I	+	-	-	-	1.579	6.842	5	36	C*03:03
106	LARKGRISV	PKP2	chr12:33049457	S70I	+	-	-	-	6.316	3.684	384	113	B*08:01
107	TYLPSAWNF	PRDM10	chr11:129780385	S1050F	+	-	-	-	0.526	14.737	17	4592	A*24:02
108	TYLPSAWNFF	PRDM10	chr11:129780385	S1050F	+	-	-	-	0.526	0.526	11	15	A*24:02
109	YLPSAWNFF	PRDM10	chr11:129780385	S1050F	+	-	-	-	1.316	2.105	27	50	A*24:02
									8.947	3.158	139	37	B*15:01
110	FRDTNGHCWV	PTP4A2	chr1:32374462	C134W	+	-	+	+	4.211	2.632	163	79	C*07:01
111	DYHAFLILI	PTPLB	chr3:123219401	Y215H	+	+	+	+	2.368	1.842	156	116	A*24:02
112	FDYHAFLI	PTPLB	chr3:123219401	Y215H	+	+	+	+	8.947	12.105	56	98	C*03:03
113	FSFDYHAF	PTPLB	chr3:123219401	Y215H	+	+	+	+	0.526	1.053	30	9	B*15:01
									3.158	3.158	9	36	C*03:03
114	FSFDYHAFL	PTPLB	chr3:123219401	Y215H	+	+	+	+	10.000	15.526	82	194	C*07:01
115	FSFDYHAFLI	PTPLB	chr3:123219401	Y215H	+	+	+	+	2.105	3.158	62	103	C*07:01
									10.000	7.368	63	39	C*03:03
116	NFSFDYHAF	PTPLB	chr3:123219401	Y215H	+	+	+	+	3.947	3.158	119	110	A*24:02
117	SFDYHAFLI	PTPLB	chr3:123219401	Y215H	+	+	+	+	4.211	4.211	357	314	A*24:02
118	YHAFLILI	PTPLB	chr3:123219401	Y215H	+	+	+	+	2.105	25.263	86	2623	C*07:01
119	YHAFLILIMI	PTPLB	chr3:123219401	Y215H	+	+	+	+	4.737	43.158	172	4633	C*07:01
120	SRSARVKPL	RAD51AP1	chr12:4668116	L322S	+	+	+	+	3.158	1.579	172	39	B*08:01
121	LVWSASDY	RANBP2	chr2:109398839	T3006S	+	-	-	-	8.421	11.579	190	328	B*15:01
122	ISSVPYAPF	RAPGEF1	chr9:134501462	P500S	+	+	-	+	2.632	52.632	42	9210	B*15:01
									7.105	15.263	36	324	C*03:03
123	SSVPYAPF	RAPGEF1	chr9:134501462	P500S	+	+	-	+	2.105	33.158	44	17443	B*15:01

124	STAPISSVPY	RAPGEF1	chr9:134501462	P500S	+	+	-	+	1.053	1.579	74	144	A*01:01
125	LGYSYVKL	RFC1	chr4:39306527	E673K	+	+	+	+	5.789	5.263	24	21	C*03:03
126	LSLDIQCEEL	RNH1	chr11:502146	Q6L	-	+	+	+	5.789	22.632	22	371	C*03:03
127	MSLDILSL	RNH1	chr11:502146	Q6L	-	+	+	+	6.316	4.211	26	14	C*03:03
128	MTFNNETF	RPA1	chr17:1782603	S285F	+	+	+	+	4.211	61.579	72	15289	B*15:01
									5.789	52.105	308	5957	C*07:01
129	MTFNNETFVM	RPA1	chr17:1782603	S285F	+	+	+	+	4.737	2.632	167	78	C*07:01
130	IYQRANDDL	RRAGC	chr1:39321435	H196Y	+	+	+	+	4.211	54.211	356	14196	A*24:02
131	TLKIQLQSCL	SATB2	chr2:200245205	S160L	+	-	+	+	5.000	61.053	231	13047	B*08:01
132	SETPANSLLL	SEZ6L2	chr16:29896934	P335S	+	+	+	+	7.895	7.895	396	401	C*07:01
133	RAKPSAPVVL	SIRPB1	chr20:1552659	S153L	+	-	-	-	6.842	63.684	32	21515	C*03:03
134	LWVGTWWVGF	SLCO4A1	chr20:61291801	A309T	+	-	-	-	1.316	1.053	79	60	A*24:02
135	LTQADCLALY	SNRNP48	chr6:7601643	R161C	+	-	+	+	0.789	1.316	38	44	A*01:01
									5.000	6.316	29	48	B*15:01
136	TQADCLAL	SNRNP48	chr6:7601643	R161C	+	-	+	+	7.895	8.947	183	209	B*15:01
137	TQADCLALY	SNRNP48	chr6:7601643	R161C	+	-	+	+	4.737	4.211	67	61	B*15:01
138	HHASNPNNPL	SOBP	chr6:107955319	S424N	-	+	+	+	5.263	4.211	209	162	C*07:01
139	RACLGSLHEL	STON1-GTF2A1L	chr2:48809593	Q607H	+	-	+	+	7.895	7.368	42	35	C*03:03
140	NISEVSTSF	SYNE1	chr6:152697530	L3104F	+	+	+	+	6.842	50.000	63	3119	B*15:01
141	TSFQKIQEF	SYNE1	chr6:152697530	L3104F	+	+	+	+	8.947	27.895	238	2781	B*15:01
									5.526	31.579	224	1903	C*07:01
142	TSFQKIQEFL	SYNE1	chr6:152697530	L3104F	+	+	+	+	7.368	17.895	316	1443	C*07:01
143	HPHERLSYL	TCF4	chr18:52937187	P106L	+	-	-	-	4.211	53.684	76	7061	B*08:01
144	LHPHERLSYL	TCF4	chr18:52937187	P106L	+	-	-	-	2.632	55.789	84	9441	C*07:01
145	PHERLSYL	TCF4	chr18:52937187	P106L	+	-	-	-	5.789	60.000	286	14739	C*07:01
146	SYLSHSSADI	TCF4	chr18:52937187	P106L	+	-	-	-	7.105	3.421	385	242	A*24:02
147	FQSPNPSTHV	TET2	chr4:106156478	S460F	+	-	-	-	6.316	10.000	263	621	C*07:01
148	LLPNENPSSY	TMEM171	chr5:72424330	P252S	+	-	+	+	1.316	2.632	22	32	B*15:01
149	PSSYYSIFNY	TMEM171	chr5:72424330	P252S	+	-	+	+	2.105	30.263	310	19577	A*01:01
150	SSYYSIFNY	TMEM171	chr5:72424330	P252S	+	-	+	+	6.842	53.684	108	6225	B*15:01
151	ITKADETSTY	TRAF7	chr16:2218084	G49E	+	-	+	-	6.842	6.842	27	21	B*15:01
152	MVSHGFSPSL	TRIM56	chr7:100730610	S6F	+	+	+	+	6.316	4.211	25	15	C*03:03
153	SHGFSPSL	TRIM56	chr7:100730610	S6F	+	+	+	+	5.263	1.579	275	44	C*07:01
154	LLFFGGQF	TRMT11	chr6:126314924	L32F	+	+	+	+	1.053	5.263	35	101	B*15:01
155	LLLFFGGQF	TRMT11	chr6:126314924	L32F	+	+	+	+	9.474	15.789	150	382	B*15:01
156	FLNMRGGLSL	UQCRC2	chr16:21982908	G245S	+	-	+	-	0.526	2.895	102	653	B*08:01
									8.684	44.474	166	1382	B*15:01
									7.895	13.158	43	104	C*03:03
157	LSLSGAKANY	UQCRC2	chr16:21982908	G245S	+	-	+	-	6.842	15.263	65	559	B*15:01
158	NMRGGLSL	UQCRC2	chr16:21982908	G245S	+	-	+	-	10.000	20.000	234	950	B*15:01
159	SLSGAKANY	UQCRC2	chr16:21982908	G245S	+	-	+	-	7.368	16.316	114	222	B*15:01
160	MAAGSSCGHI	WDR36	chr5:110439905	P310S	+	+	+	+	4.211	5.263	14	19	C*03:03
161	LQKSQVPA	ZHX1	chr8:124266911	I426L	+	-	-	-	5.789	11.053	117	282	B*15:01
162	LQKSQVPAA	ZHX1	chr8:124266911	I426L	+	-	-	-	6.842	12.632	101	254	B*15:01
163	VAGVPSQNNL	ZHX1	chr8:124266911	I426L	+	-	-	-	5.789	24.737	21	455	C*03:03

164	RIYTGEKPY	ZNF470	chr19:57089724	H643Y	+	+	+	+	5.789	8.421	90	114	B*15:01
165	RERDRHSPV	ZNF608	chr5:123977032	F1455V	+	+	+	+	6.842	6.316	379	351	B*08:01
166	RHSPVGQRHL	ZNF608	chr5:123977032	F1455V	+	+	+	+	1.053	1.053	36	36	C*07:01
167	FSSLTRHM	ZNF791	chr19:12738677	L112F	-	+	+	+	7.895	31.053	48	852	C*03:03
168	KAFMRFSSL	ZNF791	chr19:12738677	L112F	-	+	+	+	0.526	1.053	9	25	B*08:01
169	MRFSSLTRHM	ZNF791	chr19:12738677	L112F	-	+	+	+	0.526	1.053	9	36	C*07:01
170	FHLGFETL	ZNF804A	chr2:185802585	P821L	+	-	-	-	6.316	28.421	29	674	C*03:03
									1.053	1.579	38	40	C*07:01
171	FHLGFETLEL	ZNF804A	chr2:185802585	P821L	+	-	-	-	0.526	0.526	24	25	C*07:01
172	GRFHLGFETL	ZNF804A	chr2:185802585	P821L	+	-	-	-	6.316	6.316	247	265	C*07:01
173	RFHLGFETL	ZNF804A	chr2:185802585	P821L	+	-	-	-	6.842	6.316	268	187	A*24:02
174	RRRKRGRFHL	ZNF804A	chr2:185802585	P821L	+	-	-	-	0.789	37.632	195	15097	B*08:01
									3.158	58.421	101	12467	C*07:01
-	IHFMKAKGI	BZW2	chr7:16737778	insertion (frame shift)	+	+	+	+	9.650	n/a	47	n/a	C*07:01
-	IHFMKAKGIL	BZW2	chr7:16737778	insertion (frame shift)	+	+	+	+	5.600	n/a	45	n/a	C*07:01
-	LSEDCGSLL	BZW2	chr7:16737778	insertion (frame shift)	+	+	+	+	2.950	n/a	340	n/a	C*07:01
-	GSADAGGRY	CHRFAM7A	chr15:30665280	deletion (frame shift)	-	+	+	-	0.700	n/a	239	n/a	A*01:01
-		CHRNA7	chr15:32449874	deletion (frame shift)	-	+	+	-					
-	VLVLRRLVL	CHRFAM7A	chr15:30665280	deletion (frame shift)	-	+	+	-	0.400	n/a	82	n/a	B*08:01
-		CHRNA7	chr15:32449874	deletion (frame shift)	-	+	+	-					
-	WVLVLRRLVL	CHRFAM7A	chr15:30665280	deletion (frame shift)	-	+	+	-	0.500	n/a	449	n/a	B*08:01
-		CHRNA7	chr15:32449874	deletion (frame shift)	-	+	+	-					
-	ALMTSRHQSW	MST1	chr3:49723111	deletion (frame shift)	+	+	+	+	2.400	n/a	442	n/a	B*15:01
-	FCRTQMGIAM	MST1	chr3:49723111	deletion (frame shift)	+	+	+	+	0.600	n/a	308	n/a	C*03:03
-	IAMGPGATR	MST1	chr3:49723111	deletion (frame shift)	+	+	+	+	1.500	n/a	19	n/a	C*03:03
-	IAMGPGATRW	MST1	chr3:49723111	deletion (frame shift)	+	+	+	+	0.300	n/a	15	n/a	C*03:03
-	ISGVPSCAWL	MST1	chr3:49723111	deletion (frame shift)	+	+	+	+	1.700	n/a	379	n/a	C*03:03
-	RASISAGGL	MST1	chr3:49723111	deletion (frame shift)	+	+	+	+	1.550	n/a	33	n/a	C*03:03
-	SACGIGRASI	MST1	chr3:49723111	deletion (frame shift)	+	+	+	+	4.400	n/a	220	n/a	C*03:03

-	TTVPCDAAL	MST1	chr3:49723111	deletion (frame shift)	+	+	+	+	0.650	n/a	9	n/a	C*03:03
-	TTVPCDAALM	MST1	chr3:49723111	deletion (frame shift)	+	+	+	+	2.300	n/a	49	n/a	C*03:03
-	FLQFRTHTT	PTEN	chr10:89717607	deletion (frame shift)	+	+	+	+	2.200	n/a	414	n/a	B*08:01
-	FRTHTTGRQV	PTEN	chr10:89717607	deletion (frame shift)	+	+	+	+	0.200	n/a	42	n/a	C*07:01
-	FSGGTCSGL	PTEN	chr10:89717607	deletion (frame shift)	+	+	+	+	3.450	n/a	95	n/a	C*03:03
-	HTTGRQVHVL	PTEN	chr10:89717607	deletion (frame shift)	+	+	+	+	5.500	n/a	469	n/a	C*07:01
-	LPAKGEDIFL	PTEN	chr10:89717607	deletion (frame shift)	+	+	+	+	6.600	n/a	479	n/a	C*03:03
-	LQFRTHTTG	PTEN	chr10:89717607	deletion (frame shift)	+	+	+	+	1.900	n/a	104	n/a	B*15:01
-	TRSQVEPLF	WDR73	chr15:85186876	deletion (in-frame)	+	+	+	+	1.250	n/a	449	n/a	C*07:01
